# Metabolic Enzyme SLC27A5 Regulates PIP4K2A pre‐mRNA Splicing as a Noncanonical Mechanism to Suppress Hepatocellular Carcinoma Metastasis

**DOI:** 10.1002/advs.202305374

**Published:** 2023-12-07

**Authors:** Dan Nie, Xin Tang, Haijun Deng, Xiaojun Yang, Junji Tao, Fengli Xu, Yi Liu, Kang Wu, Kai Wang, Zhechuan Mei, Ailong Huang, Ni Tang

**Affiliations:** ^1^ Key Laboratory of Molecular Biology for Infectious Diseases (Ministry of Education) Institute for Viral Hepatitis Department of Infectious Diseases The Second Affiliated Hospital Chongqing Medical University Chongqing 400010 China; ^2^ Department of Gastroenterology The Chongqing Hospital of Traditional Chinese Medicine Chongqing Academy of Traditional Chinese Medicine Chongqing 400016 China; ^3^ Department of Gastroenterology The Second Affiliated Hospital Chongqing Medical University Chongqing 400016 China

**Keywords:** alternative splicing, HCC, nonmetabolic functions, PIP4K2A‐S, SLC27A5

## Abstract

Solute carrier family 27 member 5, a key enzyme in fatty acid transport and bile acid metabolism in the liver, is frequently expressed in low quantities in patients with hepatocellular carcinoma, resulting in poor prognosis. However, it is unclear whether SLC27A5 plays non‐canonical functions and regulates HCC progression. Here, an unexpected non‐canonical role of SLC27A5 is reported: regulating the alternative splicing of mRNA to inhibit the metastasis of HCC independently of its metabolic enzyme activity. Mechanistically, SLC27A5 interacts with IGF2BP3 to prevent its translocation into the nucleus, thereby inhibiting its binding to target mRNA and modulating *PIP4K2A* pre‐mRNA splicing. Loss of SLC27A5 results in elevated levels of the PIP4K2A‐S isoform, thus positively regulating phosphoinositide 3‐kinase signaling via enhanced p85 stability in HCC. SLC27A5 restoration by AAV‐*Slc27a5* or IGF2BP3 RNA decoy oligonucleotides exerts an inhibitory effect on HCC metastasis with reduced expression of the PIP4K2A‐S isoform. Therefore, PIP4K2A‐S may be a novel target for treating HCC with SLC27A5 deficiency.

## Graphical abstract

A mechanism is proposed that SLC27A5 plays a noncanonical role in inhibiting HCC progression. SLC27A5 inhibits the conversion from PIP4K2A‐L to PIP4K2A‐S via interaction with IGF2BP3, and suppresses the PI3K/AKT pathway. These findings highlight PIP4K2A‐S is a potential marker of diagnosis and prognosis for HCC. IGF2BP3 RNA decoy is a promising therapy for HCC patients with SLC27A5 deficiency.

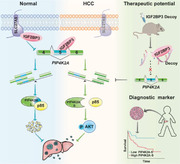

## 1 Introduction

Metabolism can be intricately linked to multiple malignant biological processes through aberrantly regulated metabolic enzymes and their metabolic products during disease progression. Thus, metabolic reprogramming plays a key role in cancer metastasis.^[^ ^1^ ^]^ Recent studies indicate that some metabolic enzymes in tumor cells acquire non‐canonical functions and directly regulate gene expression via protein‐protein interactions, protein modifications, or protein kinase activity to promote cancer progression.^[^ ^2^, ^3^, ^4^, ^5^ ^]^ For instance, UDP‐glucose 6‐dehydrogenase interacts with Hu antigen R and increases *Snail* mRNA stabilization to promote lung cancer metastasis.^[^ ^6^ ^]^ In addition, NAD(P)H quinone oxidoreductase‐1 binds to SREBP1 and promotes the progression and metastasis of hepatocellular carcinoma (HCC).^[^ ^7^ ^]^ Therefore, understanding these noncanonical roles of metabolic enzymes in cancer can provide novel strategies for treating neoplasms.

Solute carrier family 27 member 5 (SLC27A5, also known as FATP5), which is exclusively expressed in the liver, is involved in fatty acid transport and bile acid metabolism.^[^ ^8^ ^]^ SLC27A5 has been demonstrated to function as a novel tumor suppressor in HCC and is positively correlated with HCC prognosis.^[^ ^9^, ^10^ ^]^ Further, SLC27A5 deficiency can activate the NRF2/TXNRD1 pathway by increasing lipid peroxidation in HCC.^[^ ^11^ ^]^ Nevertheless, whether SLC27A5 plays non‐metabolic‐specific functions and regulates HCC progression remains unclear.

Alternative splicing (AS) is a pivotal biological process for regulating gene expression and generating proteomic diversity.^[^ ^12^ ^]^ Aberrant AS events provide potential molecular hallmarks for malignancies.^[^ ^13^, ^14^ ^]^ Accumulating evidence indicates that some RNA binding proteins (RBPs), which contain abnormal splicing factors (SFs), promote cancer progression via AS with the generation of tumor‐dependent variants.^[^ ^15^, ^16^, ^17^, ^18^, ^19^ ^]^ Dysregulation of splicing variants in many types of cancer is strongly associated with tumor invasion, metastasis, and poor prognosis.^[^ ^16^, ^20^ ^]^ Therefore, clarifying the functional impact of AS variants, their regulators, and the associated signaling pathways is critical for evaluating the effects of aberrant splicing isoform expression involved in hepatocarcinogenesis and/or metastasis.^[^ ^21^, ^22^ ^]^

As a significant RBP, insulin like growth factor 2 mRNA binding protein 3 (IGF2BP3) exhibits infrequent nuclear translocation and is involved in mRNA processing.^[^ ^23^, ^24^, ^25^ ^]^ Additionally, it contributes to cell proliferation and metastasis in various cancers.^[^ ^26^, ^27^, ^28^ ^]^ Our study identified IGF2BP3 as a new potential SLC27A5‐interacting protein in hepatoma cells. We performed high‐throughput RNA sequencing combined with RIP sequencing and verified a SLC27A5/IGF2BP3‐modulated AS event of *PIP4K2A* pre‐mRNA involving in HCC metastasis. This variant of PIP4K2A‐S (lack of exon 5) may serve as a potential prognostic marker; further, targeting mRNA alternative splicing might be a promising strategy for HCC therapy.

## 2 Results

### 2.1 SLC27A5 Directly Binds to IGF2BP3 Proteins

**Figure 1:**
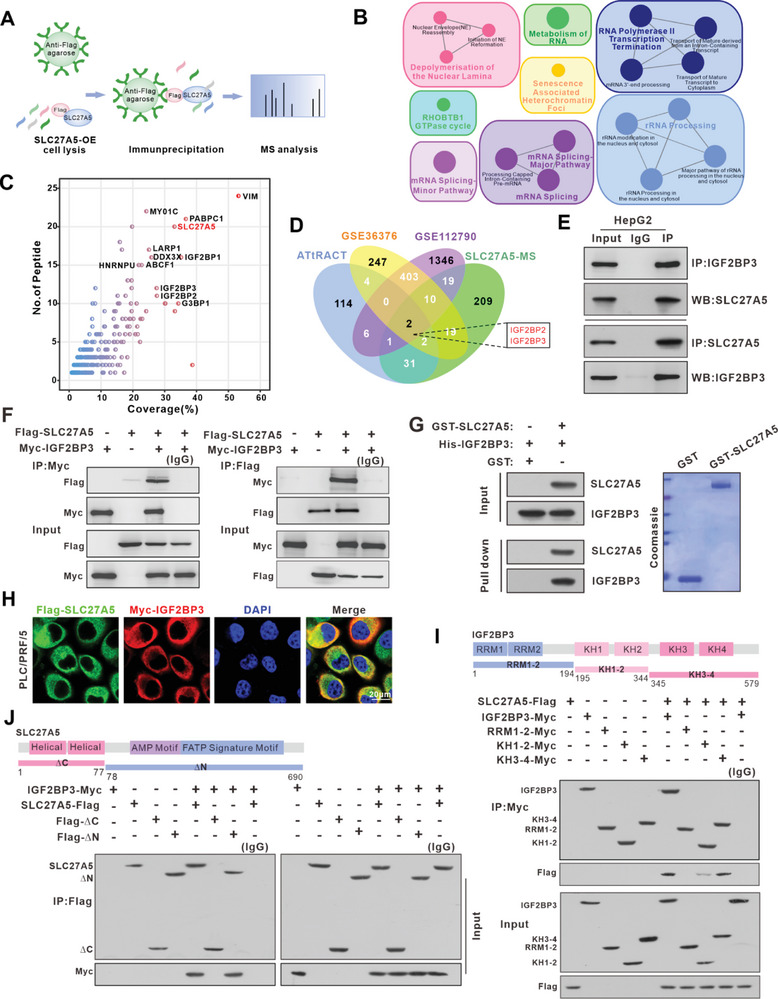
SLC27A5 binds directly to IGF2BP3 in hepatocellular carcinoma. A) Schematic of IP‐MS analysis for the SLC27A5‐interaction proteins. B) Based on ClueGO‐enrichment analysis with a two‐sided hypergeometric test, the potential SLC27A5‐interaction proteins were significantly enriched in RNA splicing and mRNA processing. The p value was adjusted using the Bonferroni method and *p* <0.05 was considered to indicate significant enrichment. C) Potential SLC27A5‐ interaction proteins obtained from IP‐MS; the most relevant proteins fall in the first quadrant, proteins with coverage (%) ≥20 and peptide count ≥10 were marked. D) SLC27A5‐interaction proteins discovered by IP‐MS, RNA‐binding proteins (RBPs) obtained from the ATtRACT database, and HCC‐associated metastasis genes acquired from the GSE database (GSE36376, GSE112790) are illustrated by Venn diagram. E) IP and WB assays of SLC27A5 with IGF2BP3 in HepG2 cells. F) Co‐IP assay was performed with anti‐Myc (left) or anti‐Flag (right) in PLC/PRF/5 cells transfected with Flag‐SLC27A5 and Myc‐IGF2BP3. Immunoblotting with anti‐Flag or anti‐Myc was performed to analyze the immunoprecipitates. lgG served as a control. G) GST pull‐down assays showing interaction between the GST‐tagged SLC27A5 and 6xHis‐IGF2BP3. GST was used as a negative control. The left panel is the western blot showing the levels of GST‐tagged SLC27A5 and 6xHis‐IGF2BP3. The right panel shows Coomassie blue staining of GST protein and GST‐tagged SLC27A5 used for the experiment. H) Subcellular colocalization of SLC27A5 and IGF2BP3 in PLC/PRF/5 cells was determined by immunofluorescence staining. (Scale bar: 20 µm). I,J) Images of the full‐length forms and mutant variants of IGF2BP3 and SLC27A5. Truncated mutants of IGF2BP3, comprising amino acids (aa) 1–194, 195–344, or 345–579, were designated as RRM1‐2, KH1‐2, and KH3‐4, respectively (I). Truncated mutants of SLC27A5, comprising amino acids (aa) 1–77 or 78–690, were designated as ΔC and ΔN, respectively(J). Co‐IP assays of Interactions between SLC27A5 and the full‐length IGF2BP3, RRM1‐2, KH1‐2, or KH3‐4 in PLC/PRF/5 cells (I). Co‐IP assays of interactions between IGF2BP3 and the full‐length SLC27A5, ΔN, or ΔC in PLC/PRF/5 cells (J).

To detect potential SLC27A5‐interacting proteins in HCC, we performed immunoprecipitation mass spectrometry (IP‐MS) (**Figure** **1**A). The results indicated that 294 proteins, including 36 RNA‐binding proteins (RBPs) were likely to interact with SLC27A5. ClueGO‐enrichment analysis revealed that these potential interacting proteins primarily played a role in regulating RNA‐related biological processes (Figure 1B,C). Additionally, our findings indicated that SLC27A5 exerted inhibitory effects on HCC cell metastasis in vitro (Figure S1A–E, Supporting Information). To identify metastasis‐associated RBPs that may interact with SLC27A5, we performed a combined analysis of the metastatic datasets of liver cancer (GSE36376, GSE112790), the RBP database (available at http://attract.cnic.es) and SLC27A5‐interacting proteins. Venn diagram analysis indicated that SLC27A5 may play an important role in promoting HCC metastasis through interaction with IGF2BP2 and/or IGF2BP3 (Figure 1D). Subsequently, co‐immunoprecipitation (Co‐IP) confirmed the interaction between SLC27A5 and IGF2BP3 (Figure 1E,F), whereas the interaction between SLC27A5 and IGF2BP2 was relatively weak (data not shown). Meanwhile, SLC27A5 and IGF2BP3 interactions were confirmed in vitro using the purified proteins (Figure 1G). Confocal analysis also indicated that SLC27A5 and IGF2BP3 were colocalized in the cytoplasm of PLC/PRF/5 and HepG2 cells (Figure 1H; Figure S2A, Supporting Information).

To verify whether the interaction between SLC27A5 and IGF2BP3 depends on its enzyme activity, we constructed an inactivated mutant of SLC27A5 (S296A),^[^ ^29^, ^30^ ^]^ and IP analysis confirmed that their interaction was not affected by enzyme activity (Figure S2B, Supporting Information). To further map the interaction sites of SLC27A5 with IGF2BP3, we generated three truncated forms of IGF2BP3 (Figure 1I) and found that the C‐terminal region of IGF2BP3 (195–579aa), particularly in the KH3‐4 domain (345‐579aa), showed a strong interaction with SLC27A5. Moreover, the IGF2BP3‐interacting domain was retained in △N of SLC27A5 (78‐ 690aa) (Figure 1J).

To further investigate the impact of the SLC27A5 enzyme inactivating mutant (S296A) on its biological function, we evaluated the proliferation and metastasis ability of HCC cells expressing the wild‐type (WT) or S296A mutant SLC27A5. The results showed that, compared to the WT SLC7A5, the S296A mutant exhibited weaker ability to suppress cell proliferation and migration in HCC cells (Figures S3A–E, Supporting Information). Taken together, these results indicate that SLC27A5 can suppress liver cancer progression by interacting with IGF2BP3, independent of its enzyme activity.

### 2.2 SLC27A5 Interacts with IGF2BP3 to Regulate *PIP4K2A* Pre‐mRNA Splicing

**Figure 2:**
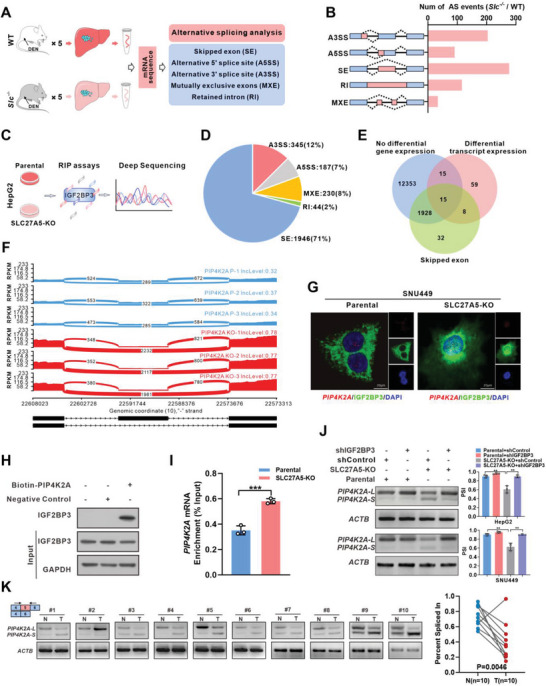
SLC27A5 interacts with IGF2BP3 to regulate PIP4K2A splicing. A) Workflow of RNA‐seq and subsequent mRNA splicing analysis for WT/*Slc27a5^−/−^* mouse liver tumors. B) Pre‐mRNA alternative splicing events regulated by SLC27A5 were analyzed by RNA splicing analysis. A3SS, alternative 3′ splicing site; SE, skipping exon; RI, retention intron; MXE, mutually exclusive exon; A5SS, alternative 5′ splicing site. C) Schematic showing that the IGF2BP3‐recognized mRNA discovered by RIP‐seq was analyzed by mRNA splicing analysis in SLC27A5‐knockout (SLC27A5‐KO) cells. D) All pre‐mRNA splicing events identified in RNA splicing analysis. E) IGF2BP3‐recognized mRNA splicing skipping events identified by RIP‐seq in SLC27A5‐KO cells. F) Splicing variants of PIP4K2A differentially display exon skipping events in the parental (Blue) and SLC27A5‐KO (Red) cells. Sashimi plots of the exon 5 junction from the aligned RNA‐seq data; the supported junction reads were marked between exons. G) IF and RNA FISH assays of IGF2BP3 and *PIP4K2A* mRNA in parental or SLC27A5‐KO SNU449 cells. IF/FISH revealed co‐localization of *PIP4K2A* mRNA (red), IGF2BP3 protein (green), and DAPI (blue)‐stained nuclei (Scale bars: 20 µm). H) RNA pull‐down assays using biotin‐labeled *PIP4K2A* pre‐mRNA. RNAs with random sequences were included as the negative control. Precipitated IGF2BP3 proteins were examined by immunoblotting. I) RIP assay of *PIP4K2A* mRNA recognized by IGF2BP3 in parental or SLC27A5‐KO SNU449 cells. J) PSI of PIP4K2A were analyzed using agarose gel electrophoresis of PCR products in SLC27A5‐KO HepG2 cells and SNU449 cells transfected with PIP4K2A‐S shRNA or negative control shRNA. Gel densitometry was analyzed using Image J to calculate the percentage‐splice‐in (PSI = splice_in/(splice_in+splice_out)) (n = 3, ^*^ *p* <0.05; ^**^ *p* <0.01). (K) PIP4K2A variants in ten pairs of tissues from patients with HCC. Gel densitometry was analyzed using Image J to calculate the PSI; the relative PSI of the variants in each sample pair was quantified (n = 10, two‐tailed, paired t‐test used for analysis) and the average PSI in ten pairs is shown (n = 10, blue column indicates matched normal tissues [N]) and red column indicates tumor [T].

As a prominent RBP, upon translocated to the nucleus and occupying the relevant binding sites, IGF2BP3 plays a crucial role in regulating the alternative splicing, and stability of target mRNA.^[^ ^24^, ^25^, ^27^, ^31^ ^]^ We identified that SLC27A5 interacted with IGF2BP3 in cytoplasm, whereas depletion of SLC27A5 promoted the nuclear translocation of IGF2BP3 (Figure S4A,B, Supporting Information). To further explore the role of SLC27A5 deletion in liver cancer metastasis, we performed RNA sequencing (RNA‐seq) on invasive liver cancer tissues in WT mice and *Slc27a5^−/‐^* mice. The results revealed that there were only 65 differentially expressed genes but 722 differential alternative splicing (AS) events occurring in *Slc27a5^−/‐^* mice (**Figure** **2A,B**; Figure S5A–C, Supporting Information). Therefore, we postulated that SLC27A5 may modulate mRNA splicing events by interacting with IGF2BP3 during HCC progression.

Next, we performed RNA immunoprecipitation followed by sequencing (RIP‐seq) and confirmed that SLC27A5 regulates AS via IGF2BP3 (Figure 2C,D). Among the key AS genes, we noted that the skipping exon was the most common event, accounting for 71% (Figure 2D). Therefore, we combined the non‐differentially expressed genes, differential transcripts, and AS events of skipping exons from RIP‐seq data for analysis. We found that 15 mRNAs may serve as splicing targets for the SLC27A5/IGF2BP3 complex (Figure 2E). Additionally, KEGG analysis revealed that the binding mRNAs of the SLC27A5/IGF2BP3 complex were mainly enriched in the phosphatidylinositol signaling pathway (Figure S5D, Supporting Information). This pathway is involved in almost all aspects of cell death and fate by regulating the activity of phosphoinositide kinases, including phosphoinositide 3‐kinases (PI3Ks) and phosphatidylinositol phosphate kinases (PIPKs).^[^ ^32^, ^33^, ^34^ ^]^ Among these 15 mRNAs, only PIP4K2A was involved in regulating the phosphatidylinositol signaling pathway. Therefore, we focused on PIP4K2A, a potential target for treating cancer and inflammation, as it catalyzes the production of PI(4.5)P2 and activates the PI3K signaling pathway.^[^ ^35^, ^36^ ^]^ Furthermore, RNA‐seq splicing analysis of SLC27A5‐KO HepG2 cells was performed, and the Sashimi plot indicated that the exon skipping occurred at the exon 5 junctions of PIP4K2A. Meanwhile, SLC27A5‐KO obviously decreased the exon 5 reads but elevated the reads across exons 4 and 6 (Figure 2F), indicating that SLC27A5 deletion was necessary for exon 5 skipping of PIP4K2A.

To determine whether SLC27A5 regulates *PIP4K2A* splicing via interaction with IGF2BP3, RNA‐fluorescence in situ hybridization (RNA FISH) along with a protein immunofluorescence (IF) assay were performed. We found that *PIP4K2A* mRNA was strongly co‐localized with IGF2BP3 protein in the nucleus of SLC27A5‐KO cells (Figure 2G; Figure S5E, Supporting Information). Conversely, binding between *PIP4K2A* mRNA and IGF2BP3 was significantly weakened in parental cells (Figure 2G; Figure S5E, Supporting Information). In addition, the interaction between *PIP4K2A* mRNA and IGF2BP3 was also validated by RNA pulldown assay (Figure 2H). Moreover, RIP‐qPCR confirmed that SLC27A5 deficiency enhanced the binding of IGF2BP3 to *PIP4K2A* mRNA (Figure 2I). The results suggested that SLC27A5 deletion may promote IGF2BP3 translocation into the nucleus and its binding with *PIP4K2A* mRNA.

To investigate the impact of the SLC27A5/IG2BP3 complex on *PIP4K2A* pre‐mRNA alternative splicing, we utilized RT‐qPCR and western blot assays to assess the expression levels of both PIP4K2A‐L (including exon 5) and PIP4K2A‐S (lacking exon 5). PIP4K2A‐L was upregulated in MHCC‐97H and PLC/PRF/5 cells overexpressing SLC27A5 (Figure S6A, Supporting Information), whereas PIP4K2A‐S was the predominant isoform in SLC27A5‐KO HepG2 and SNU449 cells and in the liver tumor tissues of *Slc27a5^−/−^* mice (Figure 2J; Figure S6B–D, Supporting Information). Furthermore, knockdown of IGF2BP3 partially decreased PIP4K2A‐S expression in SLC27A5‐KO cells (Figure 2J; Figure S6B,E, Supporting Information). Therefore, these data demonstrated that SLC27A5 regulated the splicing of *PIP4K2A* pre‐mRNA via interaction with IGF2BP3.

Finally, to investigate whether regulation of *PIP4K2A* splicing by SLC27A5 was dependent on its enzymatic activity, we transfected the S296A mutant plasmid of SLC27A5 into MHCC‐97H or PLC/PRF/5 cells and examined the splicing of *PIP4K2A* pre‐mRNA. The results showed that S296A mutant exhibited the ability of regulating *PIP4K2A* splicing similar to the wild type of SLC27A5 (Figure S6F,G, Supporting Information). In conclusion, regulation of SLC27A5 on *PIP4K2A* pre‐mRNA alternative splicing through IGF2BP3‐interaction was independent of its enzymatic activity.

### 2.3 PIP4K2A‐S Promotes HCC Progression In Vitro and In Vivo

**Figure 3:**
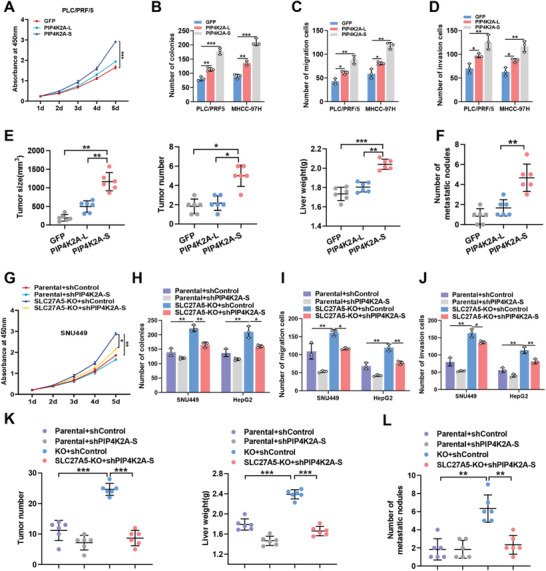
PIP4K2A‐S significantly promotes the proliferation and migration of hepatoma cells. A–D) Cell Counting Kit‐8 (CCK8) (A), colony formation (B), transwell migration (C), and invasion assays (D) for PLC/PRF/5 and MHCC‐97H cells infected with AdPIP4K2A‐L, AdPIP4K2A‐S, or AdGFP. Data are expressed as the mean ± SD for three independent experiments. ^*^ *p* <0.05; ^**^ *p* <0.01.E,F) To establish a lung metastasis model of nude mice, MHCC‐97H cells with PIP4K2A‐L or PIP4K2A‐S overexpression were orthotopically injected into their left liver lobe. After 8 weeks post‐injection, the livers and lungs were collected. Graphs (mean ± SD) showing tumor size, tumor numbers, liver weight (E), and metastatic nodules numbers (F). n = 6 (^*^ *p* <0.05; ^**^ *p* <0.01; ^***^ *p* <0.001). G–J), CCK8 (G), colony formation (H), migration (I), and invasion assays (J) for SLC27A5‐KO HepG2 cells and SNU449 cells transfected with PIP4K2A‐S shRNA or negative control shRNA. Data are expressed as the mean ± SD for three independent experiments. ^*^ *p* <0.05; ^**^ *p* <0.01. K,L) SLC27A5‐KO or parental SNU449 cells with concomitant knockdown of PIP4K2A‐S were injected into nude mice via the tail vein. Graphs (mean ± SD) showing tumor number, liver weight (K). and number of metastatic nodules (L). n = 6 (^**^ *p* <0.01; ^***^ *p* <0.001).

To investigate the biological functions of PIP4K2A‐L and PIP4K2A‐S, *PIP4K2A* mRNA splicing was validated in ten pairs of adjacent and HCC tissues (Figure 2K; Figure S6H, Supporting Information). Our data indicated that PIP4K2A‐S was enriched in HCC tissues, suggesting that PIP4K2A‐S expression may functionally contribute to HCC progression. We then examined the expression levels of the two PIP4K2A isoforms (PIP4K2A‐L and PIP4K2A‐S) in HCC cell lines (Figure S7A, Supporting Information). The results indicated that PIP4K2A‐S was highly expressed in SNU449 and HepG2 cells. Since the endogenous PIP4K2A‐L and PIP4K2A‐S expression levels were low in PLC/PRF/5 and MHCC‐97H cells, we overexpressed PIP4K2A‐L and PIP4K2A‐S in these cells (Figure S7B, Supporting Information). The results showed that PIP4K2A‐S, but not PIP4K2A‐L, promoted cell proliferation, colony formation, migration, and invasion (**Figure** **3**A–D; Figure S7C–F, Supporting Information). Importantly, PIP4K2A‐S also significantly enhanced hepatocarcinogenesis (Figure 3E; Figure S7G, Supporting Information) and lung metastasis (Figure 3F; Figure S7H, Supporting Information) in the orthotropic HCC mouse model. Notably, knockdown of PIP4K2A‐S markedly restored cell growth and cell metastasis in SLC27A5‐KO HepG2 and SNU449 cells (Figure 3G–J; Figure S8A–H, Supporting Information). Moreover, PIP4K2A‐S knockdown significantly inhibited hepatocarcinogenesis (Figure 3K; Figure S8I, Supporting Information) and lung metastasis (Figure 3L; Figure S8J, Supporting Information) in nude mice bearing SLC27A5‐KO tumor xenografts. Overall, the aforementioned results suggest that PIP4K2A‐S may serve as an oncogenic driver in HCC progression.

### 2.4 PIP4K2A‐S Stabilizes p85 Expression and Activates PI3K‐AKT Signaling

**Figure 4:**
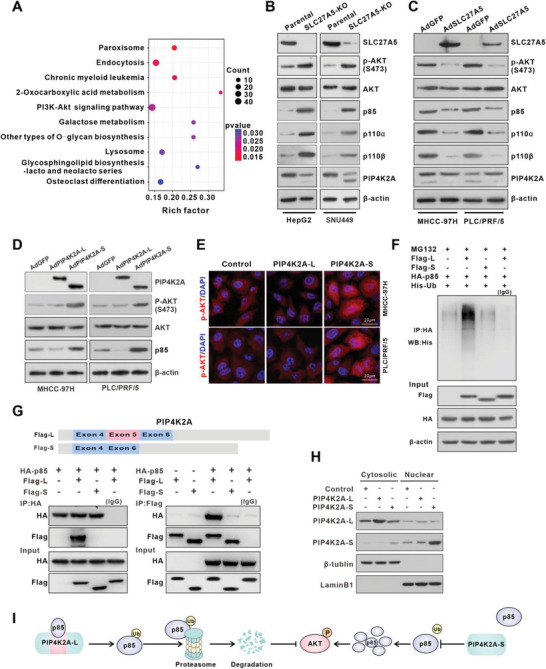
PIP4K2A‐S positively regulates PI3K signaling by stabilizing p85. A) KEGG pathway enrichment analysis of IGF2BP3‐binding genes in SLC27A5‐KO cells. B,C) Western blot analysis of the PI3K complex (p85, p110α, and p110β), phosphorylated‐AKT (p‐AKT), AKT, and PIP4K2A in SLC27A5‐KO (B) and SLC27A5‐OE (C) cells. β‐actin was used as a loading control. D) Western blot analysis of p85, p‐AKT, AKT, and PIP4K2A in hepatoma cells infected with AdGFP, AdPIP4K2A‐L, or AdPIP4K2A‐S. β‐actin was used as a loading control. E) Immunofluorescence staining of p‐AKT in hepatoma cells transduced with a control vector or PIP4K2A‐L‐ or PIP4K2A‐S‐expressing vector (scale bar: 20 µm). F) Effects of PIP4K2A‐L or PIP4K2A‐S on the ubiquitination of p85. PLC/PRF/5 cells were transduced with the indicated plasmids (5 µg each). Cells were lysed and the lysates were immunoprecipitated with anti‐HA. Co‐IP and immunoblot analysis were performed with the indicated antibodies. G) Co‐IP assay was performed with anti‐HA (left) or anti‐Flag (right) in PLC/PRF/5 cells transfected with Flag‐PIP4K2A‐L, Flag‐PIP4K2A‐S, and HA‐P85. lgG served as a control. H) Immunoblot analysis of nuclear and cytoplasmic fractions in PLC/PRF/5 cells transfected with PIP4K2A‐L‐Flag, PIP4K2A‐S‐Flag showing the levels of PIP4K2A‐L/S. LaminB1 and β‐tubulin served as the nuclear and cytoplasmic markers, respectively. I) Model of degradation of the p85 regulatory subunit in PI3K by PIP4K2A‐L, but not by PIP4K2A‐S.

A previous study has shown that PIP4K2A binds to P85 and promotes its ubiquitination in glioblastoma cells to inhibit the PI3K/AKT signaling pathway.^[^ ^36^ ^]^ In our results, we also discovered that the SLC27A5/IGF2BP3 complex was involved in regulating the PI3K/AKT signaling pathway (**Figure** **4**A). To verify the functional role of the SLC27A5/IGF2BP3 complex in regulating the PI3K/AKT signaling pathway through modulation of PIP4K2A pre‐mRNA alternative splicing, we first examined the expression of PI3K target genes in SLC27A5‐KO cells. The results showed that p85, p110α, p110β, and phosphorylated AKT (p‐AKT) were significantly unregulated when SLC27A5 was depleted (Figure 4B; Figure S9A, Supporting Information). Moreover, SLC27A5‐KO resutled in the conversion of PIP4K2A‐L to PIP4K2A‐S (Figure 4B). In contrast, SLC27A5 increased the expression of PIP4K2A‐L and downregulated p85, p110α, p110β, and phosphorylated AKT (p‐AKT) (Figure 4C). Interestingly, in MHCC‐97H and PLC/PRF/5 cells overexpressing PIP4K2A‐L, p85 and p‐AKT were downregulated, whereas overexpression of PIP4K2A‐S showed the opposite effects (Figure 4D,E). Moreover, we found that PIP4K2A‐L, but not PIP4K2A‐S, promoted ubiquitination‐mediated proteasomal degradation of p85 (Figure 4F; Figure S9B, Supporting Information). Notably, PIP4K2A‐L specifically bound to p85, whereas PIP4K2A‐S lacking exon 5 could not bind to p85 (Figure 4G; Figure S9C, Supporting Information). Further, colocalization of PIP4K2A‐L and p85 in the cytoplasm was evidenced by IF and western blot, whereas PIP4K2A‐S showed predominant expression in the nucleus (Figure 4H; Figure S9D, Supporting Information). These results indicated that the binding between p85 and PIP4K2A depended on exon 5. Taken together, SLC27A5 interacted with IGF2BP3 and blocked its binding to *PIP4K2A* pre‐mRNA, thereby inhibiting the alternative splicing of exon 5 in *PIP4K2A* and increasing PIP4K2A‐L expression. The up‐regulated PIP4K2A‐L bound to p85 and promote its ubiquitination, thus inhibiting activation of the PI3K/AKT signaling pathway (Figure 4I).

### 2.5 AAV‐Mediated Restoration of SLC27A5 Alleviated HCC Metastasis in *Slc27a5*‐KO Mice by Regulating *PIP4K2A* Splicing

**Figure 5:**
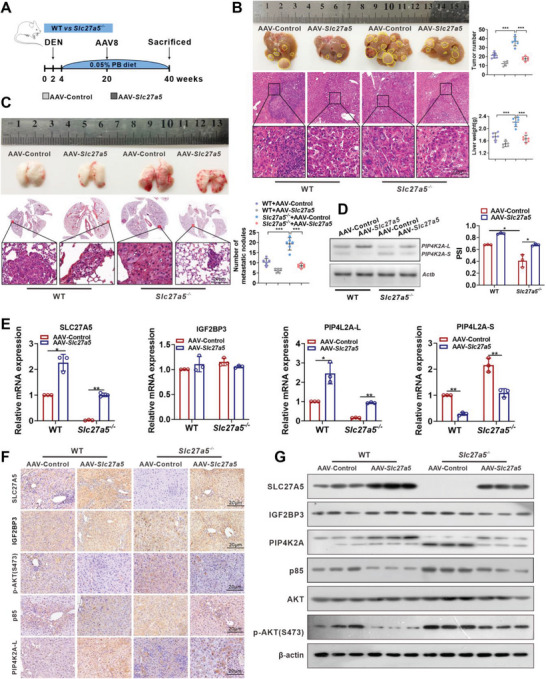
Restoration of SLC27A5 in *Slc27a5^−/−^* mice blunts HCC metastasis. A) Schematic diagram of the experimental procedures in WT and *Slc27a5^−/−^* mice. Mice were injected intraperitoneally with 75 mg kg^−1^ DEN and received phenobarbital through diet at a concentration of 0.05% for 20 weeks, followed by injection with AAV‐control or AAV‐*Slc27a5*. The liver and lung tissues were collected at 40 weeks post‐DEN/PB treatment. B,C) Gross appearances and H&E staining (scale bar: 20 µm) of samples from the livers (B) and lungs (C) with tumors (tumor nodules, n = 6/group). Representative images of tumor‐bearing livers are provided, and the tumor number and liver weight were calculated. Data indicate the mean ± SD. ^**^ *p* <0.01, one‐way analysis of variance followed by Tukey's test. D,E) qPCR assays of the indicated PIP4K2A‐L or PIP4K2A‐S mRNA expression in liver tumors. Gel densitometry analysis using Image J to calculate PSI (D). (n = 3, data are expressed as the mean ± SD, two‐tailed unpaired t test). Data are presented as the mean ± SD (E). (n = 3) ^*^ *p* <0.05. F,G) The indicated proteins in liver tumors were assessed by IHC labeling (scale bar: 20 µm) (F) and western blot (G).

In order to observe the role of SLC27A5 in liver cancer metastasis, we restored the expression of SLC27A5 in invasive liver cancer via tail vein injection of AAV8‐TBG control and AAV8‐TBG‐*Slc27a5* (n = 6) in WT or *Slc27a5^−/‐^* mice (**Figure** **5**A). AAV‐mediated restoration of hepatic SLC27A5 decreased the liver weight and tumor numbers in *Slc27a5^−/‐^* mice (Figure 5B). Moreover, the number of lung metastasis in SLC27A5‐treated mice was significantly lower than that in *Slc27a5^−/‐^* mice (Figure 5C). Consistent with our in vitro data, depletion of SLC27A5 induced partial IGF2BP3 translocation to the nucleus and caused an isoform switch of PIP4K2A‐L to PIP4K2A‐S in the liver tumors, whereas restoration of SLC27A5 in *Slc27a5^−/‐^* mice increased the PIP4K2A‐L expression but repressed PIP4K2A‐S expression (Figure 5D–G). Importantly, activation of PI3K/AKT signaling and enhancement of HCC migration were observed in *Slc27a5* ^−/−^ mice, which were reversed by restoring SLC27A5 (Figure 5F,G; Figure S9E, Supporting Information). These results indicated that SLC27A5 alleviated HCC metastasis in *Slc27a5*‐KO mice by regulating *PIP4K2A* splicing.

### 2.6 SLC27A5 Deficiency with PIP4K2A‐S Upregulation Promotes HCC Metastasis

**Figure 6:**
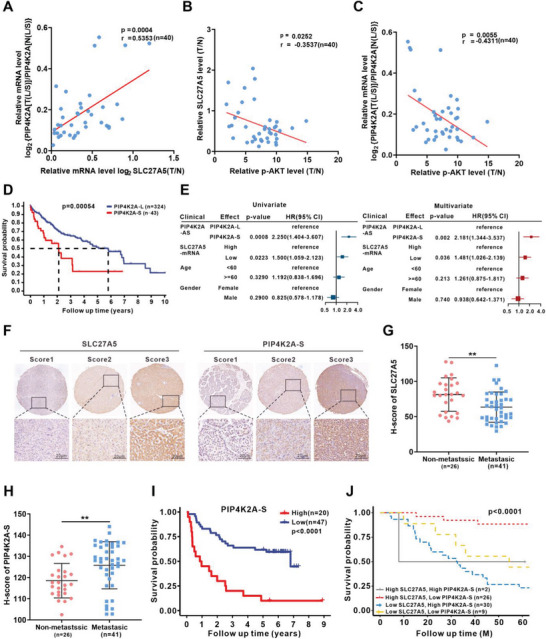
The variant PIP4K2A‐S is enriched in HCC and associated with poor prognosis. A‐C) Correlation analyses of the *PIP4K2A‐L/S* ratio and *SLC27A5* mRNA expression (A), SLC27A5 and p‐AKT expression (B), *PIP4K2A‐L/S* ratio and p‐AKT expression (C) in tumor tissues from 40 patients with HCC. Data are presented as the mean ± SEM. D) Kaplan–Meier survival analysis of overall survival of PIP4K2A‐L and PIP4K2A‐S in HCC patients from TCGA Splice Seq – liver hepatocellular carcinoma dataset (PIP4K2A‐L n = 324, PIP4K2A‐S, n = 43). Overall survival of PIP4K2A‐L and PIP4K2A‐S were calculated by Kaplan‐Meier method, and statistical significance was measured by log‐rank test. E) The univariate and multivariate Cox regression model of the clinical characteristics and *SLC27A5*‐mRNA expression levels. Univariate and multivariate Cox proportional hazards regression models were used to estimate the independent factors for overall survival. F) Representative IHC staining of SLC27A5 and PIP4K2A‐S in an HCC tissue microarray. Scores (0–3) were calculated according to the percentage of stained cells and intensity. (Scale bar: 20 µm). G,H) Staining scores of SLC27A5 and PIP4K2A‐S in tumor tissues from patients with HCC, with (n = 41) or without metastatic recurrence (n = 26). The percentage of non‐metastatic or metastatic recurrence of HCC (n = 67) was stratified by SLC27A5 and PIP4K2A‐S expression using the “survminer” R package (version 3.6.3). I) Kaplan–Meier analysis of overall survival of PIP4K2A‐S in the tissue microarray cohort (n = 67), HCC patients were grouped according to the PIP4K2A‐S expression level in tumor. J) Kaplan–Meier analysis of overall survival rate of SLC27A5 and PIP4K2A‐S in an HCC tissue microarray cohort (n = 67), stratified by SLC27A5 and PIP4K2A‐S expression.

Next, we determined the expression of SLC27A5, PIP4K2A‐L, PIP4K2A‐S, and p‐AKT in 40 pairs of HCC and adjacent tissues (Table S1, Supporting Information). Compared to adjacent tissues, both SLC27A5 expression and the PIP4K2A‐L/S ratio were downregulated in HCC tissues, whereas p‐AKT expression was significantly upregulated (Figure S10A,B, Supporting Information). Notably, a positive correlation was observed between *SLC27A5* expression and the ratio of PIP4K2A‐L/S (**Figure** **6**A), whereas SLC27A5 expression (Figure 6B) and the ratio of PIP4K2A‐L/S (Figure 6C) was negatively correlated with p‐AKT expression. Furthermore, patients with high PIP4K2A‐S expression levels displayed poorer overall survival based on data from TCGA Splice‐Seq dataset (Figure 6D). Univariate and multivariate Cox regression analysis revealed that SLC27A5 deficiency and high PIP4K2A‐S expression are independent risk factors for death (Figure 6E).

Moreover, we surveyed the expression levels of SLC27A5 and PIP4K2A‐S using a cohort of samples from patients with HCC (Table S2, Supporting Information) by performing immunohistochemistry (IHC) assays. As shown in Figure 6F, the patients were stratified by high versus low SLC27A5 and/or PIP4K2A‐S levels. SLC27A5 expression was negatively correlated with PIP4K2A‐S (Figure S10C, Supporting Information). Notably, low SLC27A5, high PIP4K2A‐S, and low SLC27A5 levels coupled with high PIP4K2A‐S levels were strongly associated with HCC metastasis (Figure 6G,H) and negatively correlated with survival duration (Figure 6I; Figure S10D,E, Supporting Information). Furthermore, patients with low SLC27A5 and high PIP4K2A‐S expression levels had the poorest survival (Figure 6J). Therefore, PIP4K2A‐S may represent an independent factor for poor prognosis and serve as a promising target for treating HCC.

### 2.7 Decoy Oligonucleotides Specifically Binding to IGF2BP3 Inhibit HCC Progression In Vivo

**Figure 7:**
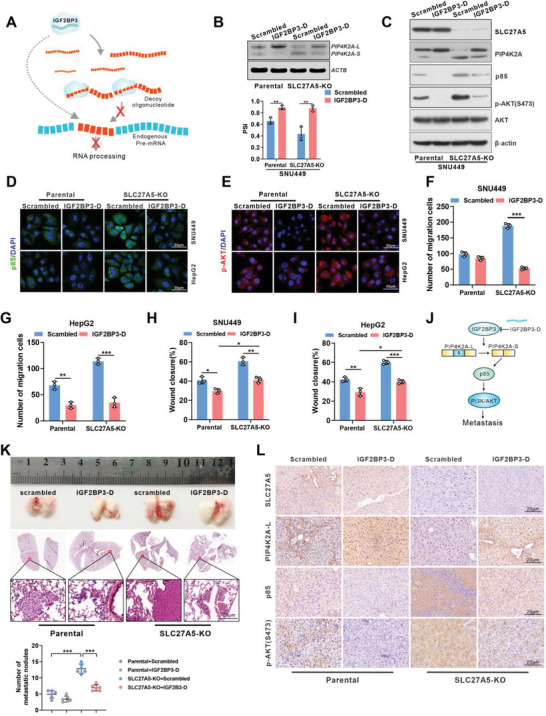
IGF2BP3 decoy oligonucleotides block SLC27A5‐induced *PIP4K2A* splicing. A) Schematic representing the inhibition of RNA binding proteins by decoy oligonucleotides. B) The PSI of PIP4K2A was analyzed using agarose gel electrophoresis of PCR products in SLC27A5‐KO SNU449 cells transfected with scrambled or IGF2BP3 decoy oligonucleotides. PSI was calculated by gel densitometry using Image J (^*^ *p* <0.05; ^**^ *p* <0.01). C – E) SLC27A5‐KO cells were treated as described in (B). Cells were subjected to western blot analysis (C) or IF staining (D and E) with the indicated proteins (scale bar: 20 µm). F – I) Effects of the scrambled or IGF2BP3 decoy oligonucleotides on cell migration and wound healing in SLC27A5‐KO cells. All data were expressed as mean ± standard deviation (x ± s), and analyzed using one‐way ANOVA for comparing between groups (n = 3). ^*^ *p* <0.05; ^**^ *p* <0.01. J) Schematic showing the IGF2BP3 RNA decoy oligonucleotides inhibiting tumor growth and metastasis. K) SLC27A5‐KO SNU449 cells transfected with scrambled or IGF2BP3 decoy oligonucleotides (scrambled, n = 6; IGF2BP3‐D, n = 6) were orthotopically injected into the left liver lobes of nude mice. After 8 weeks post‐injection, the liver and lung tissues were obtained for H&E staining. The metastatic nodules were counted (^*^ *p* <0.05; ^**^ *p* <0.01). L) The indicated proteins in liver tumors were evaluated by IHC labeling (scale bar: 20 µm).

Previous studies defined the RNA binding consensus of IGF2BP3 as GGC/CA‐ through in vitro SELEX and in vivo iCLIP‐seq approaches.^[^ ^37^ ^]^ Based on these studies, we designed RNA decoy oligonucleotides,^[^ ^38^ ^]^ containing the GGC/CA‐ sequence, to block the RNA binding activity of IGF2BP3 by interacting with the RRM domains (**Figure** **7**A). Transfection of the IGF2BP3 decoy (IGF2BP3‐D) oligonucleotides into HCC cell lines partially blocked cell proliferation and migration (Figure S11A,B, Supporting Information). Moreover, *PIP4K2A* splicing was inhibited by transfection of the IGF2BP3‐D oligonucleotide (Figure 7B,C; Figures S11C and S12A, Supporting Information). Notably, splicing of PIP4K2A lasted for at least 96 h, indicating the enduring biological effect of the decoy oligonucleotides (Figure S11C, Supporting Information). Furthermore, in SLC27A5‐KO cells, treatment with IGF2BP3‐D oligonucleotides induced an isoform switch from PIP4K2A‐S to PIP4K2A‐L, suppressed the expression of p85 and p‐AKT, and inhibited cell migration and wound healing compared to that in the scrambled group (Figure 7B–J; Figure S12A–E, Supporting Information). Importantly, IGF2BP3‐D oligonucleotides reduced lung metastasis and inhibited the liver tumor growth induced by SLC27A5 depletion (Figure 7K; Figure S12F, Supporting Information). Moreover, IHC analysis indicated that SLC27A5‐KO enhanced the expression of p85 and p‐AKT but repressed PIP4K2A‐L, whereas the IGF2BP3‐D oligonucleotide significantly increased PIP4K2A‐L expression in vivo (Figure 7L). Taken together, these data indicate that RNA decoy oligonucleotides, specifically bound to IGF2BP3, reduce the cancer‐related splicing events of PIP4K2A and inhibit tumor growth and metastasis (Figure 7J).

## 3 Discussion

**Figure 8:**
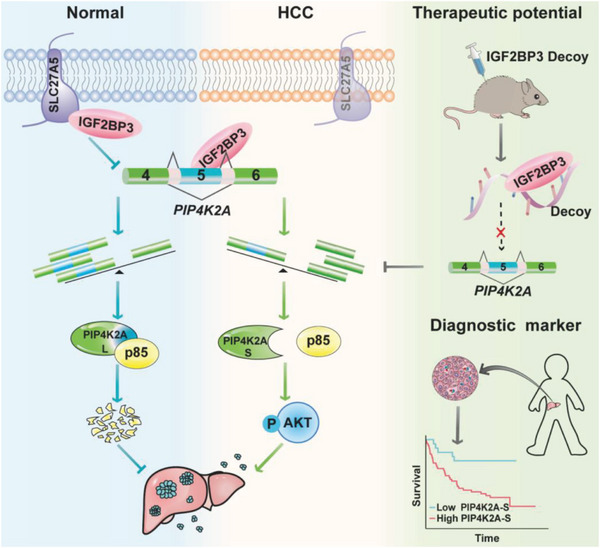
Mechanistic model: SLC27A5 plays a noncanonical role in HCC. Normally, SLC27A5 interacted with IGF2BP3. In HCC, SLC27A5 depletion induced IGF2BP3 translocation into nuclear and generated PIP4K2A‐S isoform with exon 5 skipping, which promoted cell metastasis via enhancing p85 protein stability to activate PI3K/AKT signaling pathway. PIP4K2A‐S may represent an independent factor for poor prognosis and serve as a promising target for the treatment of HCC.

SLC27A5 deficiency plays a critical role in increasing polyunsaturated lipids and promoting cancer progression.^[^ ^9^, ^11^, ^39^ ^]^ Although a few previous studies indicated that the potential role of SLC27A5 in the transcriptional regulation of gene expression does not depend on its enzyme activity,^[^ ^10^ ^]^ most studies on SLC27A5‐mediated suppression of HCC progression have focused on its enzymatic role in lipid or bile acid metabolism.^[^ ^11^, ^39^, ^40^ ^]^ In this study, we found that SLC27A5 directly interacted with IGF2BP3 to inhibit the interaction of *PIP4K2A* mRNA with IGF2BP3, leading to decreased expression of the PIP4K2A‐S variant to inhibit HCC progression (**Figure** **8**).

IGF2BPs are primarily cytoplasmic proteins; however, upon translocating to the nucleus and occupying relevant binding sites, they play a crucial role in the post‐transcriptional regulation of target mRNA.^[^ ^41^, ^42^ ^]^ Alternative splicing is a crucial process in the post‐transcriptional processing of pre‐mRNA. Previous studies show that IGF2BP3 may also play a role in regulating pre‐mRNA alternative splicing.^[^ ^25^ ^]^ The eCLIP analysis revealed that IGF2BP3 not only interacted with the 3′UTR of target transcripts but also bound to the intronic regions and splice sites of target transcripts. In leukemia, IGF2BP3 directly binds to target mRNA and is involved in regulating alternative splicing events.^[^ ^25^ ^]^ Additionally, IGF2BP3 directly binds to PKM mRNA and regulates its splicing in lung cancer.^[^ ^24^ ^]^ Consistent with previous research,^[^ ^23^ ^]^ a portion of IGF2BP3 translocates to the nucleus in the absence of SLC27A5, regulates the alternative splicing of PIP4K2A mRNA by directly binding to it, and is involved in the progression of liver cancer. In future studies, it is necessary to characterize the mechanism governing IGF2BP3 nuclear localization and the splicing function of IGF2BP3

A recent study showed that PIP4K2A physically interacted with p85 and induced proteasome‐mediated degradation, thereby regulating PI3K.^[^ ^36^ ^]^ Similarly, we revealed that PIP4K2A‐L bound to and induced p85 degradation. In contrast, PIP4K2A‐S was incapable of binding to p85 owing to the loss of exon 5, which resulted in absence of PIP4K2A‐mediated p85 ubiquitination. These results suggest that exon 5 of PIP4K2A was the binding domain for p85. Further, in other lipid kinases, the activation loop in the kinase domain determines substrate specificity and is also involved in membrane recruitment.^[^ ^43^, ^44^ ^]^ Exon 5 of PIP4K2A contains 51 amino acids, located in the ATP‐binding domain of PIP4K2A.^[^ ^32^ ^]^ Deletion of exon 5 may have altered the conformation of the activation loop and modified its subcellular localization.^[^ ^32^, ^45^ ^]^ Interestingly, we found that the subcellular distribution of PIP4K2A‐S differed from that of PIP4K2A‐L; PIP4K2A‐S was mainly distributed in the nucleus whereas PIP4K2A‐L was localized in the cytoplasm. Future studies on the mechanism underlying the nuclear accumulation of PIP4K2A‐S are required for deeper insights into the roles of PIP4K2A variants.

The results of clinical studies targeting PIP4Ks are promising, but this strategy may not be appropriate for all cancers.^[^ ^36^ ^]^ In a recent study, the selective and potent PIP4K2A inhibitors (BAY‐091 and BAY‐297) developed by Bayer did not induce an antiproliferative effect on p53 null tumor cells, suggesting that PIP4K inhibitors must potently inhibit both PIP4K2A and other PIP4K isoforms to exert an antitumor effect.^[^ ^46^ ^]^ Thus, attenuation of the antitumor effect of PIP4K2A inhibitors may be related to the splicing variants produced by alternative splicing. Here, we identified a new splicing variant, PIP4K2A‐S, which provides a promising alternative for additional PIP4K targets and may be a potential therapeutic target for HCC.

Recent studies indicate that targeting alternative mRNA splicing is a promising strategy for cancer therapy.^[^ ^13^ ^]^ Thus, various approaches have targeted AS events to inhibit the initiation of poor AS variants. One strategy is the application of decoy oligonucleotides to compete for the natural binding targets of splicing factors for reducing their activity.^[^ ^47^ ^]^ In addition, engineered U7 snRNAs and CRISPR‐based approaches for gene editing have the ability to target specific alternative splicing events.^[^ ^48^, ^49^ ^]^ RNA decoy oligonucleotides offer the following two advantages: 1) they have fewer side effects than those of complete knockdown^[^ ^47^ ^]^ and 2) they are single stranded RNA molecules, which can be delivered to the organs more efficiently than double stranded siRNA molecules.^[^ ^50^, ^51^ ^]^ In our study, we designed RNA decoy oligonucleotides targeting IGF2BP3 and used them to block splicing factor activity by interacting with the RRM domains. Treatment with IGF2BP3 RNA decoy oligonucleotides caused isoform switching of PIP4K2A‐S to PIP4K2A‐L and suppressed the expression of p85 and p‐AKT, which inhibited tumor growth and metastasis both in vitro and in vivo. These findings indicate that RNA decoy oligonucleotides targeting tumor‐specific splicing‐related proteins may be a more promising clinical strategy for treating HCC.

In summary, our findings indicate that SLC27A5 interacts with IGF2BP3, partially blocking the nuclear translocation of IGF2BP3 and its binding to PIP4K2A mRNA, thereby inhibiting conversion from PIP4K2A‐L to PIP4K2A‐S and suppressing activation of the PI3K/AKT pathway. This is an unrecognized noncanonical mechanism for SLC27A5 to inhibit tumor progression independently of its enzymatic activity. These findings highlight a potential prognostic marker for HCC and suggest that targeting IGF2BP3 activity is a promising strategy for treating patients with HCC as well as SLC27A5 deficiency.

## 4 Experimental Section

### Cell Culture and Cell Transfection

MHCC‐97H, PLC/PRF/5, HEK293, and HepG2 cells were cultured in DMEM (HyClone, Logan, UT, USA) mixed with 1% penicillin/streptomycin (HyClone) and 10% fetal bovine serum (FBS, Gibco, NY, USA) at 37 °C with 5% CO_2_. SNU449 cells were cultured in Roswell Park Memorial Institute (RPMI) 1640 medium (Gibco, Grand Island, NY, USA) mixed with 1% penicillin/streptomycin (MedChemExpress, Monmouth, NJ, USA) and 10% FBS at 37 °C with 5% CO_2_.

### Animal Studies

*Slc27a5* ^−/−^ C57 BL/6J mice were generated from crosses between *Slc27a5* ^+/‐^ mice and *Slc27a5^+/−^* mice using the CRISPR/Cas9 system (European Mouse Mutant Archive, KOCMP‐04385‐Slc27a5), and WT mice were used as controls (n = 5 per group). After treatment with DEN (75 mg kg^−1^) and PB (at a concentration of 0.05%), HCC was induced in these mice as described previously.^[^ ^52^, ^53^ ^]^ Tumor volume (V) was calculated as: V [cm^3^]  =  (length [cm])  ×  (width [cm]  ×  (width [cm])/2. Metastasized tumor nodules and foci in the lungs were counted through microscopic observation.^[^ ^16^ ^]^

For SLC27A5 overexpression, AAV8‐TBG‐control or AAV8‐TBG‐*Slc27a5* (Gene ID: 26459, NM_‐_009512.2, OBiO Technology, Corp., Ltd. Shanghai, China) was injected via tail vein into WT and *Slc27a5* ^−/‐^ mice after 20‐weeks of DEN/PB treatment (2 × 10^11^ genome copies per mouse). After 40 weeks, the mice were euthanized and their liver/lung tissues with tumors were examined.

BALB/c nude mice were randomly grouped (n = 6/group) for the orthotopic implantation model. MHCC97H cells (1 × 10^5^, AdPIP4K2A‐L‐, AdPIP4K2A‐S‐, AdGFP‐infected) were suspended in a 50 µL Matrigel (356234, BD Biosciences, CA, USA)/phosphate‐buffered saline (PBS) mixture (1:1 ratio, volol) and then implanted into the left liver lobe of each nude mouse. All mice were euthanized after 8 weeks of implantation. All animal experiments were compliant with the Medical Ethics Committee of Chongqing Medical University (reference number: 2022053).

### Clinical Specimens

Human HCC tissues and corresponding paired nontumorous liver tissues were collected from 40 patients at the Second Affiliated Hospital of Chongqing Medical University, approved by the IRB of Chongqing Medical University (reference number: 2022053).

### CRISPR/Cas9‐Mediated Gene Editing

Single guide RNA sequences were designed to target the SLC27A5 and GSR genes (refer to Table S3, Supporting Information). The sequences were designed using the E‐CRISP tool (http://www.e‐crisp.org/E‐CRISP/designcrispr.html) and subsequently cloned into the CRISPRv2 lentiviral vector expressing Cas9, which was kindly provided by Prof. Ding Xue from Tsinghua University, Beijing, China. Lentiviruses were then packaged, and stable knockout cell lines were selected following previously established protocols.^[^ ^11^ ^]^

### Plasmid Construction

The cloned human vector cDNAs for SLC27A5 were ligated into the TO4‐3Flag expression vector to produce Flag‐tagged proteins. The mutants of SLC27A5 (S296A) were constructed by site‐directed mutagenesis using wild‐type (WT) SLC27A5‐3xFlag plasmid as the template.

### Cell Transfection and Reagent Treatment

SNU449 cells (parental or SLC27A5‐KO) were transfected with 200 nm RNA decoys using Lipofectamine RNAiMAX (Thermo Fisher Scientific). SNU449 and HepG2 cells were transfected with 50 nm shRNAs using Lipofectamine 3000 (Thermo Fisher Scientific). PLC/PRF/5 and 293 cells were treated with MG132 (HY13259, MedChemExpress) for 8 h.

### IP‐MS Assay

PLC/PRF/5 cells, which were pre‐transfected with SLC27A5‐TO4‐3Flag or TO4‐3Flag plasmid, were incubated overnight at 4°C with ANTI‐FLAG M2 affinity gel (A2220, Sigma). Coomassie blue was used to stain the protein complexes. The excised protein gel bands were submitted to Shanghai Applied Protein Technology Co., Ltd. for identification of proteins that bind to SLC27A5. After undergoing reduction reaction and alkylation treatment, the samples were incubated with trypsin (mass ratio, 1:50). The digested samples were then dried and analyzed by mass spectrometry after injecting 10 or 20 µL of each sample. Mass spectrometry (MS) analysis was performed on a Lumos Tribrid Orbitrap Mass Spectrometer (Thermo Scientific) equipped with Ultimate 3000 (Thermo Scientific) nano‐high‐performance liquid chromatograph. The raw MS data were analyzed using Mascot 2.2 software to identify the immunoprecipitating proteins. Further, Gene Ontology enrichment analysis was performed using clusterProfiler package in R, with Fisher's Exact Test, and the significant p value cutoff was set at 0.05.

### RNA‐Seq Analysis for Identifying Metastasis‐Associated Splicing Events

Five cases of primary HCC tissue samples were collected from *Slc27a5^−/−^* mice and five HCC tissue samples from WT mice for RNA‐seq. rMATS were used for splicing analysis in the data set. The expression levels of alternative splicing events (ASE) in primary HCC tissues with *Slc27a5^−/−^* mice and WT mice were quantified, and the differentially expressed isoforms or exons between two groups were analyzed using MISO. The ASEs with |Δdifferent| > 0.2 and bayes_factor > 2 were considered significantly differentially spliced.

### RIP Assay

The EZ‐Magna RIP kit (17‐701, Millipore) was used to perform RIP. In short, ≈2 × 10^7^ SLC27A5‐KO HepG2 cells were lysed with 200 µL RIP Lysis Buffer at low temperature for 5 min and centrifuged. The supernatants were incubated with magnetic beads (Thermo Fisher Scientific) and the appropriate antibodies for 8 h at 4 °C with gentle rotation. The beads were washed four times with RIP lysis buffer containing RNase inhibitor (Millipore, Massachusetts, USA). The RNA was extracted by chloroform and detected by RT‐PCR. Total RNA (input controls) and negative IgG controls were detected simultaneously. Table S4 (Supporting Information) indicates the specific primers used for the RIP assay. Table S5 (Supporting Information) lists the antibodies used for RIP assay.

### Immunofluorescence Staining

After plated on coverslips in a 12‐well dish, HepG2 cells were fixed with paraformaldehyde, and then blocked using bovine serum albumin. The coverslips were then incubated with primary antibody (SLC27A5, Novus Biologicals, 1:200; IGF2BP3, Proteintech, 1:400) at 4 °C overnight. The cells were then incubated with goat anti‐rabbit IgG/TRITC or goat anti‐mouse IgG/FITC for 2 h at room temperature. The cells were viewed and photographed under a fluorescence microscope (Leica TCS SP8, Solms, Germany).

### RNA Fluorescence In Situ Hybridization Combined with Immunofluorescence

The cells were seeded on the coverslips pre‐coated with 5 µg mL^−1^ poly‐L‐ornithine (P4957, Sigma–Aldrich) for 30 min, and grown for 24 h followed by fixation using 4% paraformaldehyde. The cells were then washed thrice in PBS and permeated using 0.5% Triton X‐100 (Sigma–Aldrich). The cell were then blocked with 4% IgG‐free Bovine Serum Albumin and incubated with the primary antibody (see Table S5, Supporting Information) for 4–16 h. After washing thrice with PBS, the cells were incubated with the secondary antibody for 1 h and then fixed using 4% paraformaldehyde. After two further PBS washes, the cells were examined using a fluorescence in situ hybridization (FISH) kit (GenePharma, F16501/50); the RNA FISH probes are listed in Table S4 (Supporting Information). The cells were then washed once with water, sealed with nail polish, and photographed under a fluorescence microscope (Leica TCS SP8, Solms, Germany).

### Glutathione S‐Transferase (GST) Pull‐Down Assay

GST‐SLC27A5 was purified by glutathione agarose beads (GE Healthcare, Piscataway, NJ, USA). His‐IGF2BP3 was purified by HisPur Ni‐NTAmagneticbeads (Thermo Fisher, MA, USA). The purified proteins were incubated with glutathione agarose beads for 3 h. The beads were washed with wash buffer (137 mm NaCl, 2.7 mm KCl, 10 mm Na_2_HPO_4_, 2 mm KH_2_PO_4_ and 0.5% Triton X‐100), mixed with 2× SDS loading buffer and boiled for 8 min. The inputs/elutions were further analyzed by Coomassie staining and/or immunoblot analysis.

### RNA Pull‐Down Assays

RNA Pull‐down assays were performed by incubating 0.1 mm biotin‐PIP4K2A with purified His‐IGF2BP3 (5 µg) for 0.5 h at 25 °C. RNAs with random sequences served as the negative control. Complexes were pulled down using Pierce streptavidin agarose beads and then immunoblotted with the indicated antibodies.

### PIP4K2A‐L and PIP4K2A‐S Antibody Production

Anti‐PIP4K2A‐L or Anti‐PIP4K2A‐S antibodies generated using the NSLTRSAPLPNDSQAR and RFGIDDQDFQYIVEC antigens were purchased from ABclonal.

### Co‐Immunoprecipitation and Western Blot Analysis

Cells were resuspended in RIPA lysis buffer mixed with an EDTA‐free protease inhibitor cocktail (#C0001, TargetMol, MA, USA). The precleared lysate was incubated with magnetic beads (Thermo Fisher Scientific, Carlsbad, CA, USA) and then immunoprecipitated overnight at 4 °C using the indicated antibodies. The protein complex was then subjected to western blotting. Briefly, the samples were separated using sodium dodecyl‐sulfate polyacrylamide gel electrophoresis and then blotted on PVDF membranes (Millipore, Billerica, MA, USA). The membrane was then blocked for 30 min with 5% nonfat milk, and incubated overnight with primary antibodies. This was followed by incubation with horseradish peroxidase‐conjugated secondary antibodies. The signal was then visualized using ECL (WBKLS0500, Millipore). GAPDH was used as the loading control. The antibodies in the study were listed in Table S5 (Supporting Information).

### qPCR

TRIzol reagent (Invitrogen, Carlsbad, CA, USA) was used to extract total RNA from tissues or cells. PrimeScript RT Reagent Kit (TaKaRa, Shiga, Japan) was used for cDNA synthesis. The target gene expression was then detected using real‐time PCR (SYBR green) with specific primers. The PCR conditions were as follows: 95 °C for 3 min, followed by 40 cycles of 95 °C for 15 s, 60 °C for 15 s, and 72 °C for 20 s, concluding with an extension step of 72 °C for 5 min. The data were analyzed using the 2^−∆∆Ct^ method. The primers used were listed in Table S4 (Supporting Information).

### Statistics and Reproducibility

The dead animals and euthanized animals were excluded according to the IACUC protocol. The sample size was not predetermined by any statistical method. Investigators who were blinded to the experimental conditions did not perform the data collection and analyses. Animals were randomized and treated as former indication for each in vivo. All samples were analyzed equally with no sub‐sampling resulting and there was no need for randomization in the in vitro experiments. The experiments in Figures 2I–K; 3A–L; 5D,E; and 7B,F–I and in Figures S1A,C,D,E; S3A–E; S6A,C,F;S7C; S8B; S10B; S11A,B (Supporting Information) were performed at least thrice and similar results were obtained. The figure captions indicate the statistical analyses performed. The means ± standard error of the mean (SEM) is indicated by the error bars. Statistical calculations were performed using GraphPad Prism software (version 7). Clinicopathological characteristics of patients with HCC were analyzed using χ^2^ analysis. Data are expressed as mean ± standard deviation (SD).

### Ethics Approval Statement

This study was approved by the Research Ethics Committee of Chongqing Medical University (approval number: 2022054). All animal experiments were performed under the guidelines of the institutional Animal Care and Use Committee at Chongqing Medical University.

### Patient Consent Statement

Informed consents were obtained from all involved participants.

## Conflict of Interest

The authors declare no conflict of interest.

## Author Contributions

D.N., X.T., H.J.D. and X.J.Y. contributed equally to this work. D.N., X.T., H.J.D., X.J.Y., J.J.T., F.L.X., Y.L., and K.W. performed experiments. H.J.D. and M.Z.C. provided technical and clinical support. X.J.Y. evaluated histopathology. K.W., M.Z.C., A.L. and N.T. designed and supervised experiments. N.D., K.W., and N.T. wrote the manuscript. All authors read and approved the manuscript.

## Supporting information

Supporting Information

Supporting Information

## Data Availability

The data that support the findings of this study are available from the corresponding author upon reasonable request.
