# Supplementary material for: Metabolic Enzyme SLC27A5 Regulates PIP4K2A pre‐mRNA Splicing as a Noncanonical Mechanism to Suppress Hepatocellular Carcinoma Metastasis
Source: Adv Sci (Weinh). 2023 Dec 7;11(5):2305374. doi: 10.1002/advs.202305374 (PMC10837360; doi:10.1002/advs.202305374)
Supplement: Supplementary file 1 — Supporting Information [file ADVS-11-2305374-s002.pdf]

ADVANCED SCIENCE
Open Access

Supporting Information

for *Adv. Sci.*, DOI 10.1002/advs.202305374

Metabolic Enzyme SLC27A5 Regulates PIP4K2A pre-mRNA Splicing as a Noncanonical Mechanism to Suppress Hepatocellular Carcinoma Metastasis

Dan Nie, Xin Tang, Haijun Deng, Xiaojun Yang, Junji Tao, Fengli Xu, Yi Liu, Kang Wu, Kai Wang, Zhechuan Mei*, Ailong Huang* and Ni Tang**

Supporting Information

Metabolic enzyme SLC27A5 regulates PIP4K2A pre-mRNA splicing as a noncanonical mechanism to suppress hepatocellular carcinoma metastasis

Dan Nie, Xin Tang, Haijun Deng, Xiaojun Yang, Junji Tao, Fengli Xu, Yi Liu, Kang Wu, Kai Wang, Zhechuan Mei*, Ailong Huang*, Ni Tang**

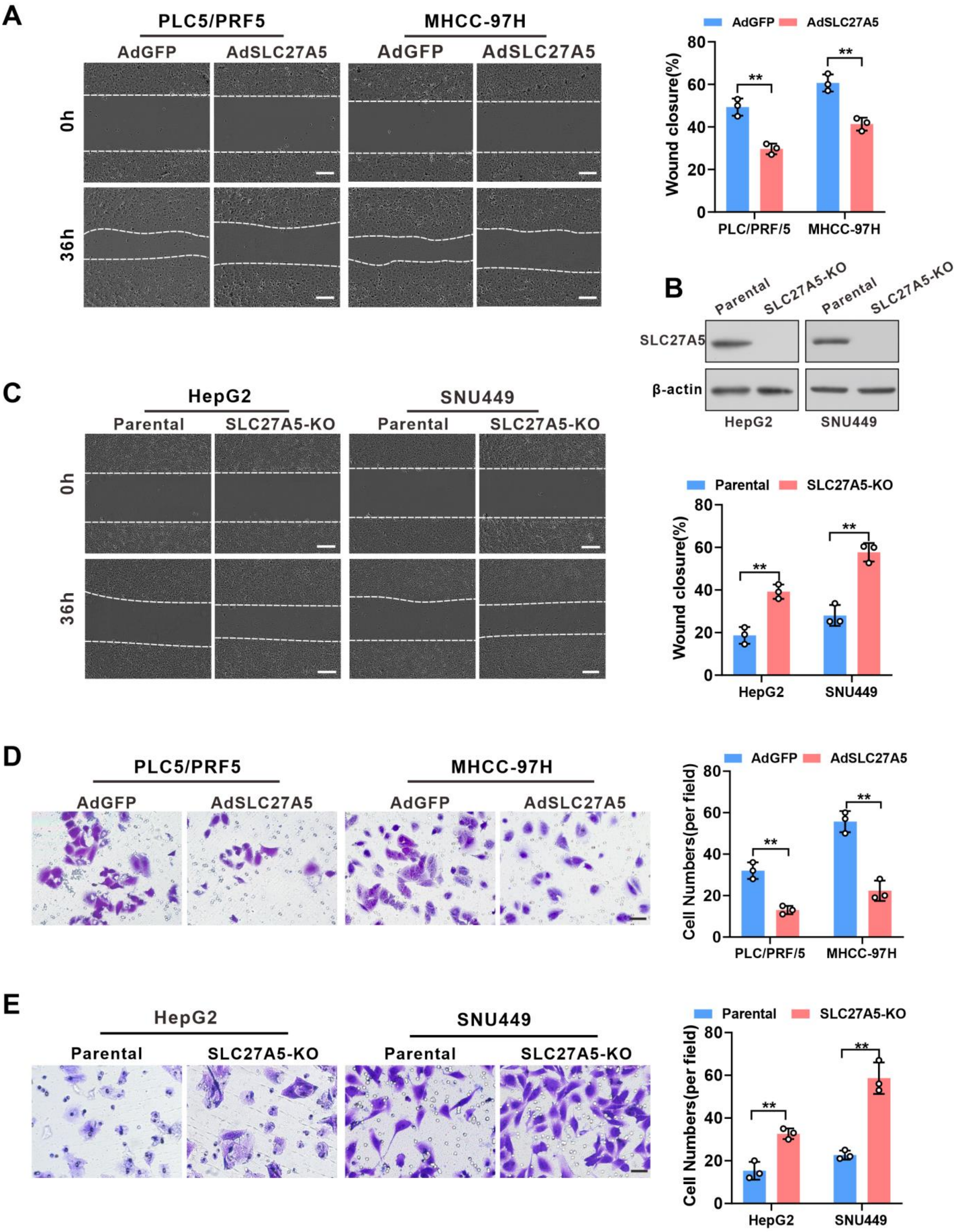

Figure S1. SLC27A5 inhibits HCC metastasis in vitro.

(A) Wound healing assay of SLC27A5-overexpressing (SLC27A5-OE) PLC/PRF/5 and MHCC-97H cells. (B) The efficiency of SLC27A5 knockout was confirmed by western blot. (C) Wound healing assays of SLC27A5-knockout (SLC27A5-KO) HepG2 and SNU449 cells. (D) Transwell assays of SLC27A5-OE PLC/PRF/5 and MHCC-97H cells. (E) Transwell

migration assays of SLC27A5-KO HepG2 and SNU449 cells. All data are presented as the mean ± SD for three independent experiments. *$p < 0.05$, **$p < 0.01$, ***$p < 0.001$.

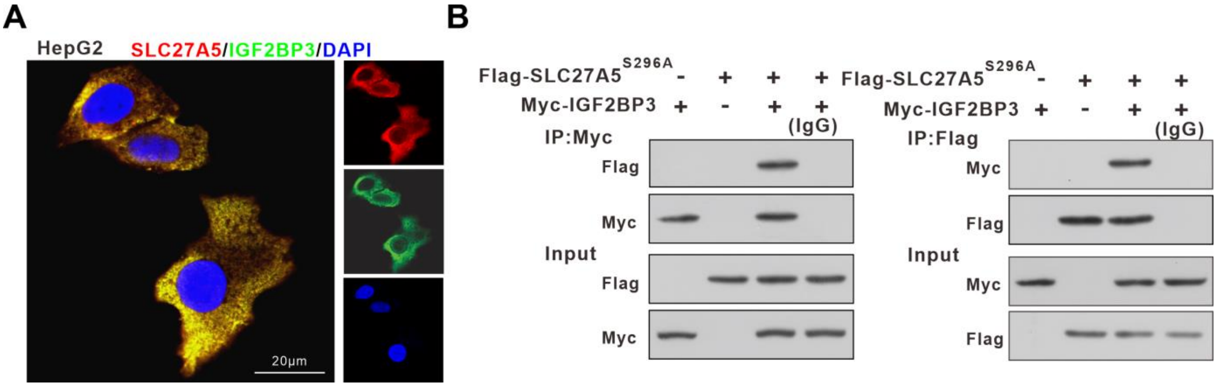

Figure S2. SLC27A5 interacts with IGF2BP3 independently of its enzymatic activity.

(A) Immunofluorescence staining showed co-localization of SLC27A5 and IGF2BP3 proteins in HepG2 cells. (B) Co-IP assay was performed with anti-Myc (left) or anti-Flag (right) in PLC/PRF/5 cells transfected with recombinant plasmids Flag-SLC27A5 (S296A) and Myc-IGF2BP3. Immunoblotting with anti-Flag or anti-Myc was applied to analyze the immunoprecipitates. lgG served as a control.

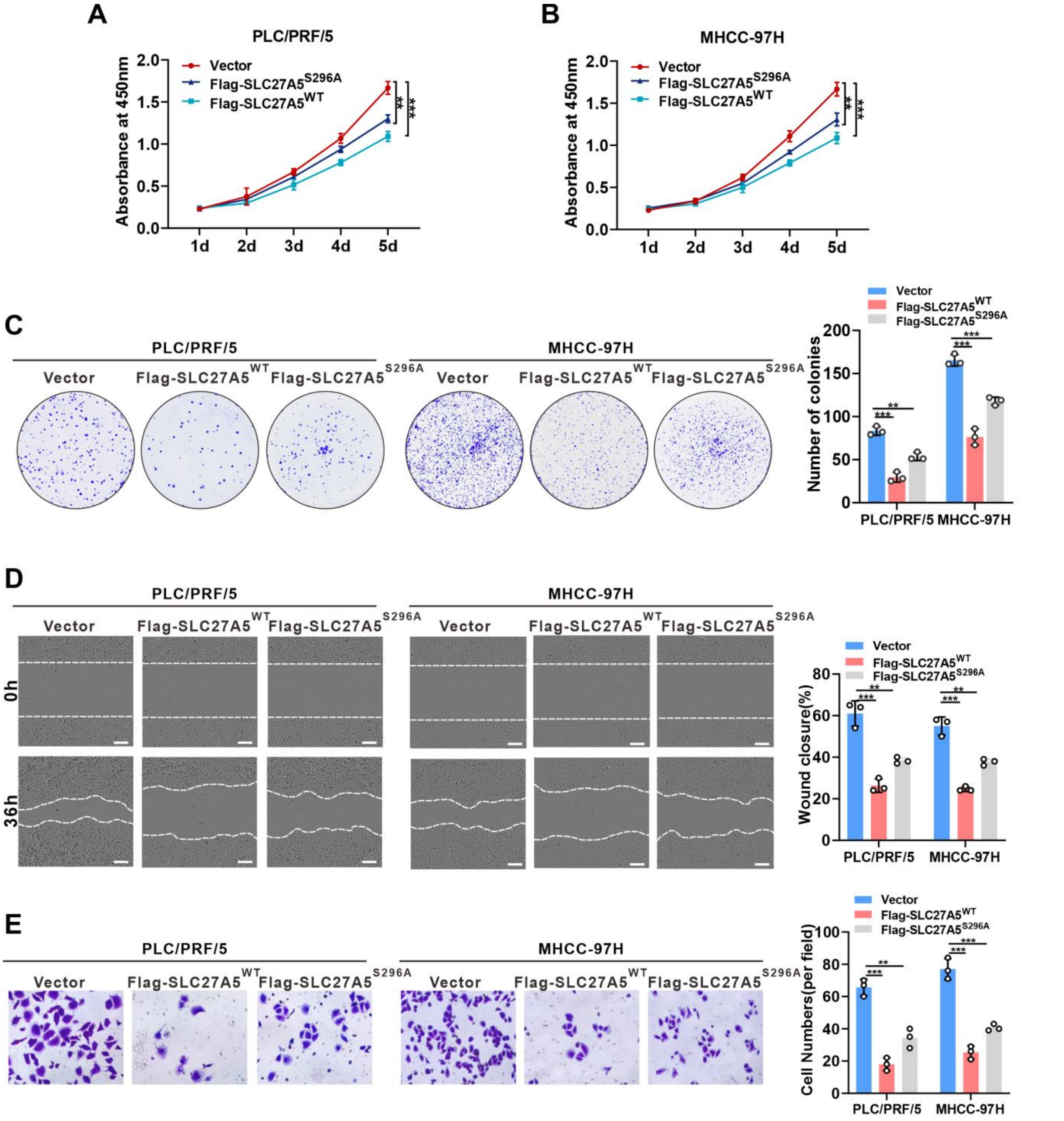

Figure S3. SLC27A5 promotes liver cancer progression independently of its enzymatic activity.

(A-E). Cell counting kit-8 (A, B), colony formation (C), wound healing (D), and transwell assays (E) of PLC/PRF/5 and MHCC-97H cells transfected with Flag-SLC27A5 (WT) or Flag-SLC27A5 (S296A). All data are presented as the mean ± SD for three independent experiments. **p < 0.01, ***p < 0.001.

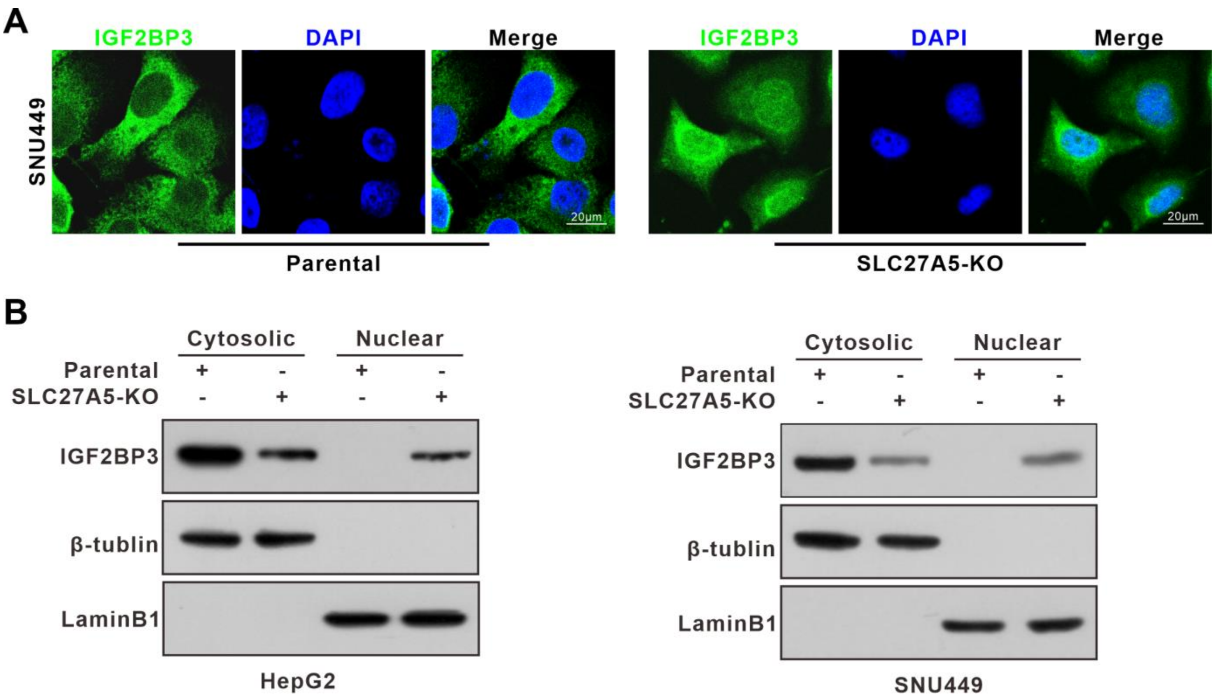

Figure S4. SLC27A5 deficiency promotes IGF2BP3 translocation to the nucleus.

(A) IF staining of IGF2BP3 in parental or SLC27A5-KO SNU449 cells. (Scale bar: 20 μm). (B) Immunoblot analysis of IGF2BP3 in the nuclear and cytoplasmic fractions of parental or SLC27A5-KO cells. LaminB1 and β-tubulin served as nuclear and cytoplasmic markers, respectively.

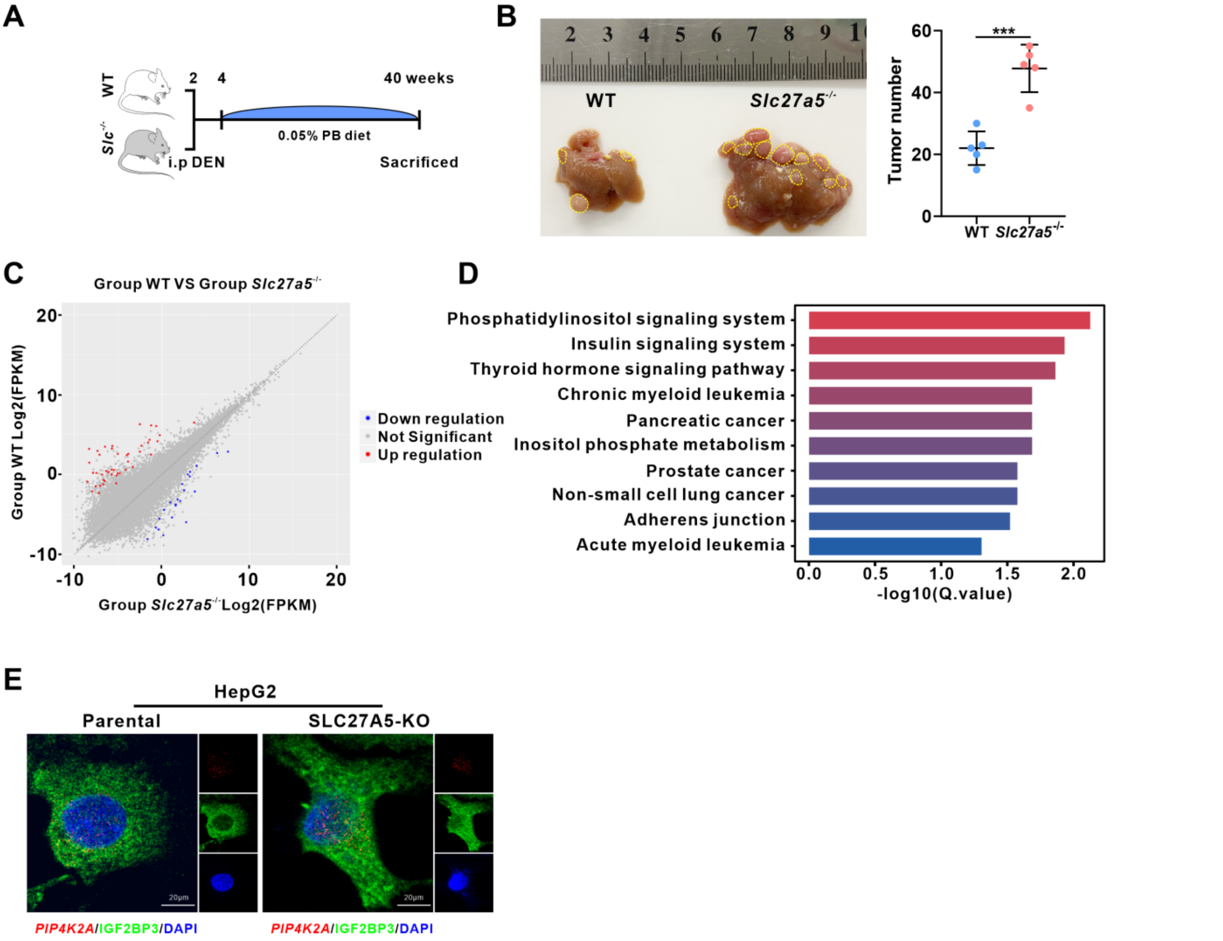

Figure S5. SLC27A5 deficiency promotes IGF2BP3 translocation to the nucleus and induces PIP4K2A mRNA splicing.

(A) A DEN/PB-induced metastatic liver cancer model was established in *Slc27a5*$^{-/-}$ and WT mice. Liver and lung tissues were collected at 40 weeks after DEN treatment. (B) Livers images and the numbers of tumor nodules, n = 5/group. Data are presented as the mean ± SD. ***$p < 0.001$, one-way ANOVA followed by Tukey's test. (C) Volcano plot showing the 35 differential genes between WT and *Slc27a5*$^{-/-}$ mice liver tumor tissues. Each gene is plotted according to its expression level (log2FC) and q-value (-log10(q-value)). Red indicates upregulation and blue indicates downregulation. FPKM indicates reads per kilobase per million mapped reads. (D) KEGG pathway enrichment of genes bound to IGF2BP3 in SLC27A5-KO cells, performed by clusterProfiler package in R, with Fisher's Exact Test; the significant p value cutoff was set at 0.05. (E) RNA FISH and IF assays of IGF2BP3 and *PIP4K2A* mRNA in SLC27A5-KO HepG2 cells.

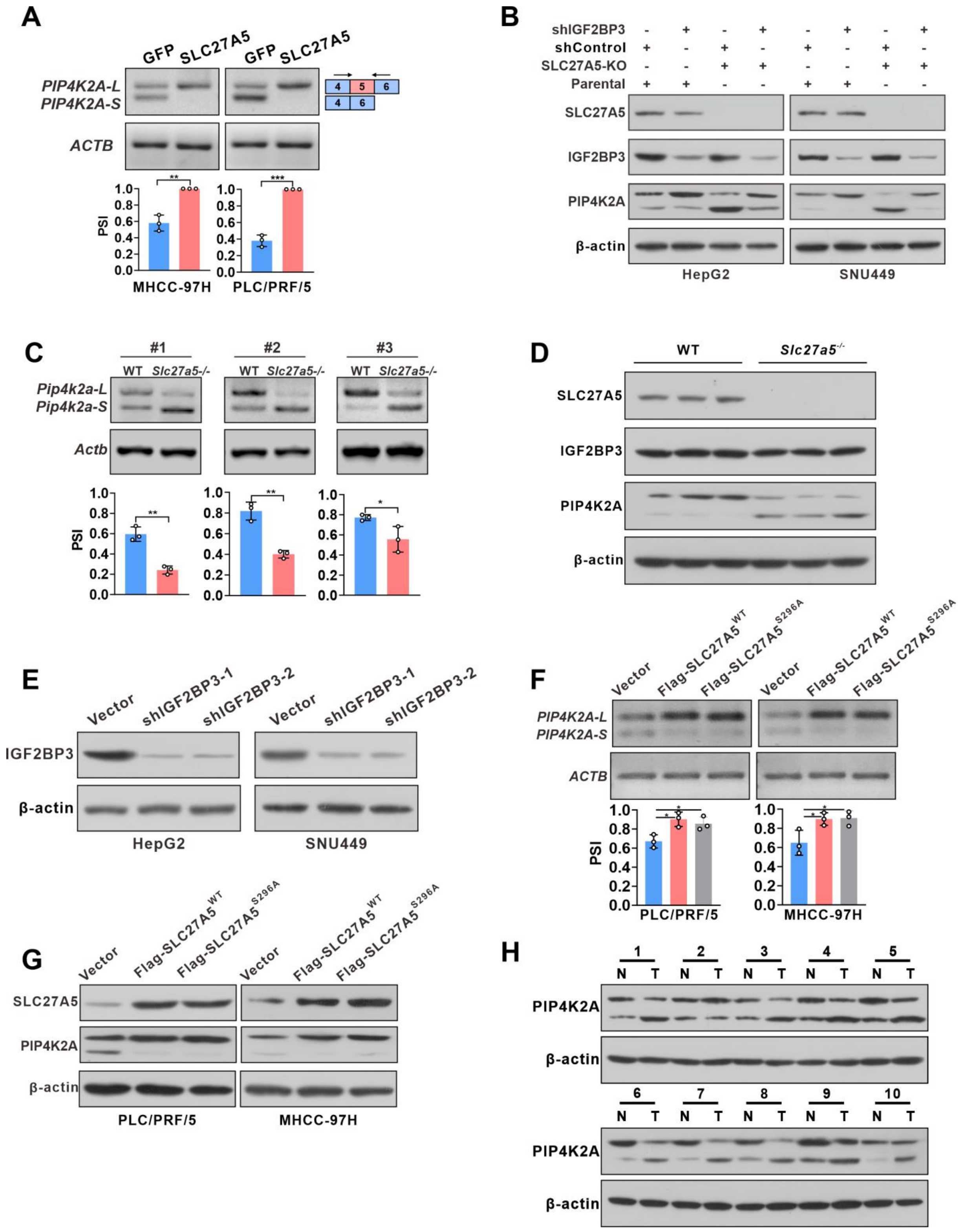

Figure S6. SLC27A5 regulates PIP4K2A pre-mRNA alternative splicing through IGF2BP3.

(A) PSI of *PIP4K2A* in SLC27A5-OE cells (n = 3, **p < 0.01). (B) Western blot analysis of PIP4K2A in SLC27A5-KO cells with IGF2BP3 knockdown. (C) PSI of PIP4K2A in the liver tumor tissues of WT and Slc27a5$^{-/-}$ mice (n = 3, *p < 0.05; **p < 0.01). (D) Western blot

analysis of PIP4K2A in the liver tumors of WT or Slc27a5$^{-/-}$ mice. (E) The IGF2BP3 knockdown efficiency was confirmed by western blot analysis. (F) PSI of *PIP4K2A* in PLC/PRF/5 and MHCC-97H cells transfected with the recombinant plasmid Flag-SLC27A5 (WT) or Flag-SLC27A5 (S296A). (G-H) Western blot analysis of PIP4K2A in cells transfected with Flag-SLC27A5 (WT) or Flag-SLC27A5 (S296A)(H) and in ten pairs of adjacent and HCC tissues from patients.

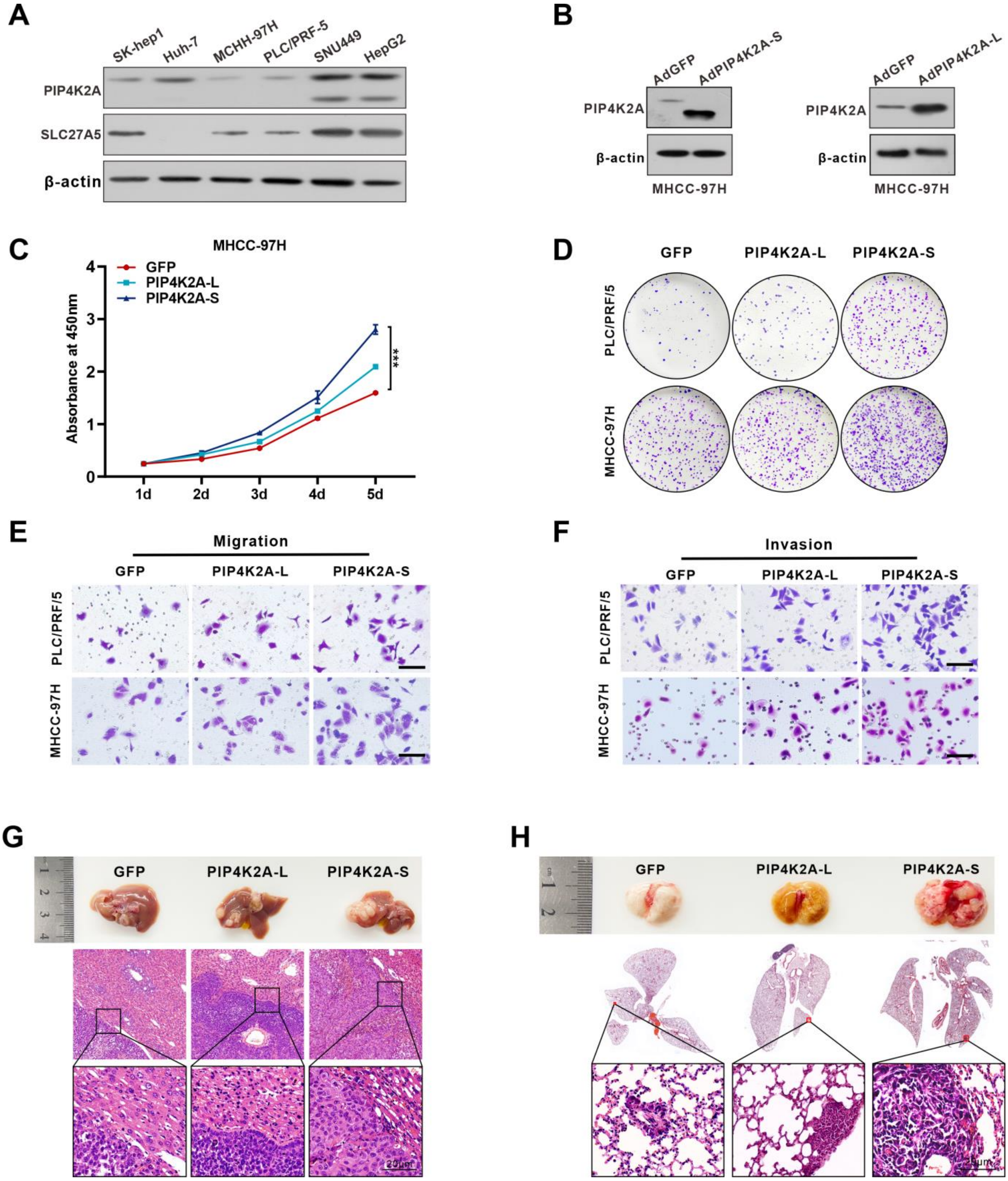

Figure S7. PIP4K2A-S plays a positive role in HCC proliferation and migration.

(A) The protein expression of SLC27A5 and PIP4K2A in HCC cells. (B) Overexpression of PIP4K2A-L and PIP4K2A-S was confirmed by western blot. (C-F) Cell counting kit-8 assays (C), colony formation assays (D), transwell migration (E), and invasion assays (F) for PLC/PRF/5 and MHCC-97H cells infected with AdPIP4K2A-L, AdPIP4K2A-S, or AdGFP. (G) Tumor images and H&E staining of liver tissues. (H) Tumor images and H&E staining of lung tissues. ***$p < 0.001$.

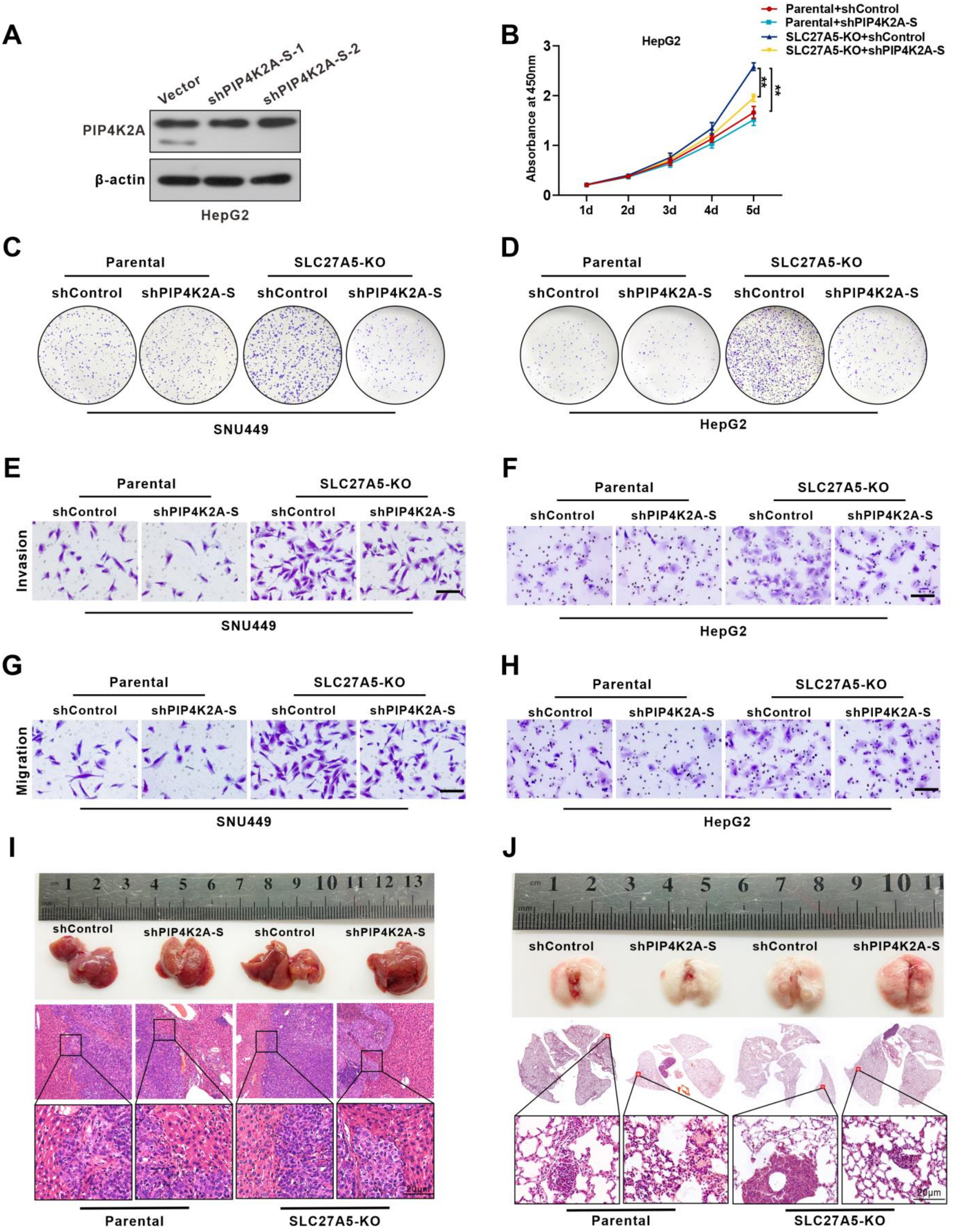

Figure S8. SLC27A5 deficiency plays a negative role in the proliferation and migration of HCC through PIP4K2A-S.

(A) PIP4K2A-S knockout efficiency was confirmed by western blot. (B-H) Cell counting kit-8 assays (B), colony formation assays (C,D), transwell migration (G), and invasion assays (H) for SLC27A5-KO HepG2 cells and SNU449 cells transfected with PIP4K2A-S shRNA or

negative control shRNA. (I - J). Typical stained images for metastatic liver (I) and lung tumors (J) (n = 6/group) were shown. *p < 0.05; **p < 0.01.

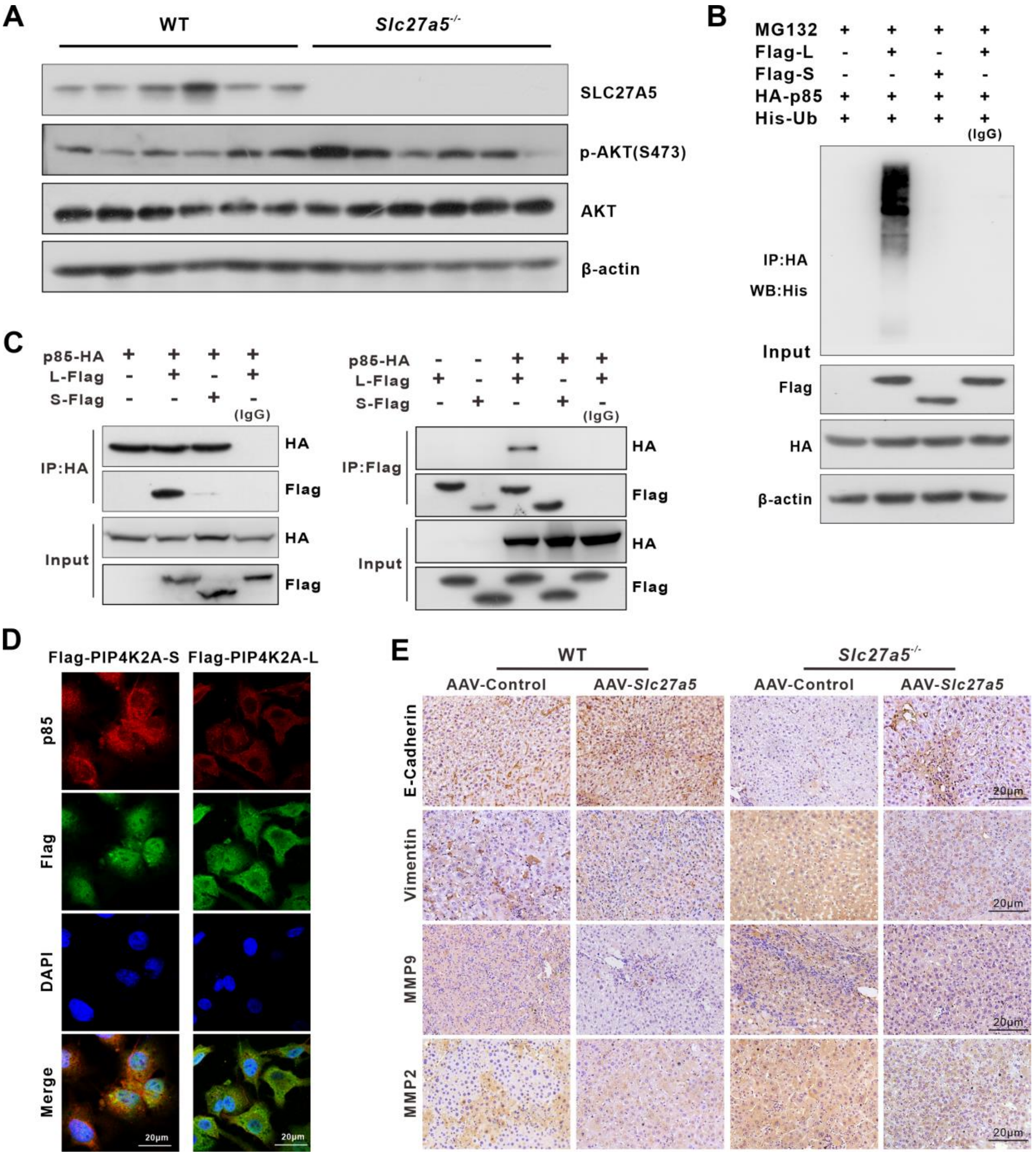

Figure S9. PIP4K2A-S inhibits p85 degradation.

(A) Protein expression of SLC27A5, p-AKT, and AKT in the liver tumors of WT or *Slc27a5*$^{-/-}$ mice. (n = 6). (B) Immunoprecipitation was performed in 293 cells co-transfected with HA-p85, His-Ubiquitin, and Flag-PIP4K2A-L or Flag-PIP4K2A-S. The ubiquitinated forms of p85 were detected by western blot with anti-His antibodies. (C) PIP4K2A-L interacts with p85. Co-IP assay was performed with anti-HA (left) or anti-Flag (right) in PLC/PRF/5 cells transfected with HA-p85 and Flag-PIP4K2A-L or Flag-PIP4K2A-S. The immunoprecipitates were analyzed with anti-Flag and anti-HA antibodies, respectively. (D) IF staining of

PIP4K2A-L/S and p85 in PLC/PRF/5 cells transfected with Flag-PIP4K2A-L or Flag-PIP4K2A-S. (Scale bars: 20 μm). (E) The indicated proteins in liver tumors were assessed by IHC staining.

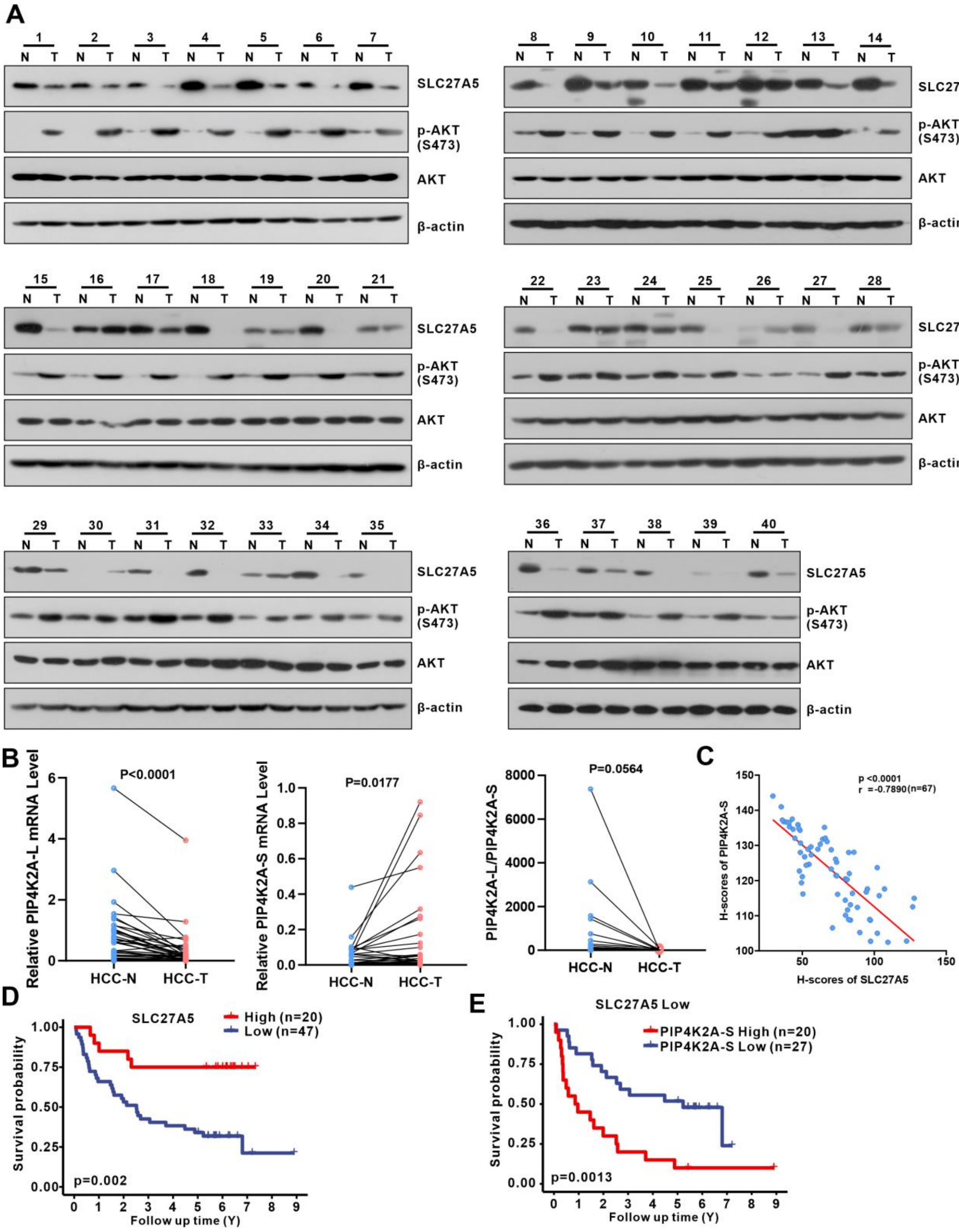

Figure S10. SLC27A2 deficiency and PIP4K2A-S overexpression were detected in HCC tissues.

(A) Immunoblot analysis of SLC27A5, p-AKT, and AKT in tumor tissues and adjacent non-tumor tissues from patients with HCC (n = 40). (B) Normalized PIP4K2A-L, PIP4K2A-S,

and PIP4K2A-L/PIP4K2A-S copy numbers in 40 pairs of HCC tissues. (C) Correlation analyses of staining scores for PIP4K2A-S and SLC27A5 tissue microarray cohorts (n = 67). Data are presented as the mean ± SEM. (D) Survival analysis of high or low SLC27A5 expression in the tissue microarray cohort (n = 67 patients with HCC). (E) Survival analysis of high or low PIP4K2A-S expression in the tissue microarray cohort (n = 67) of patients with HCC showing low expression of SLC27A5.

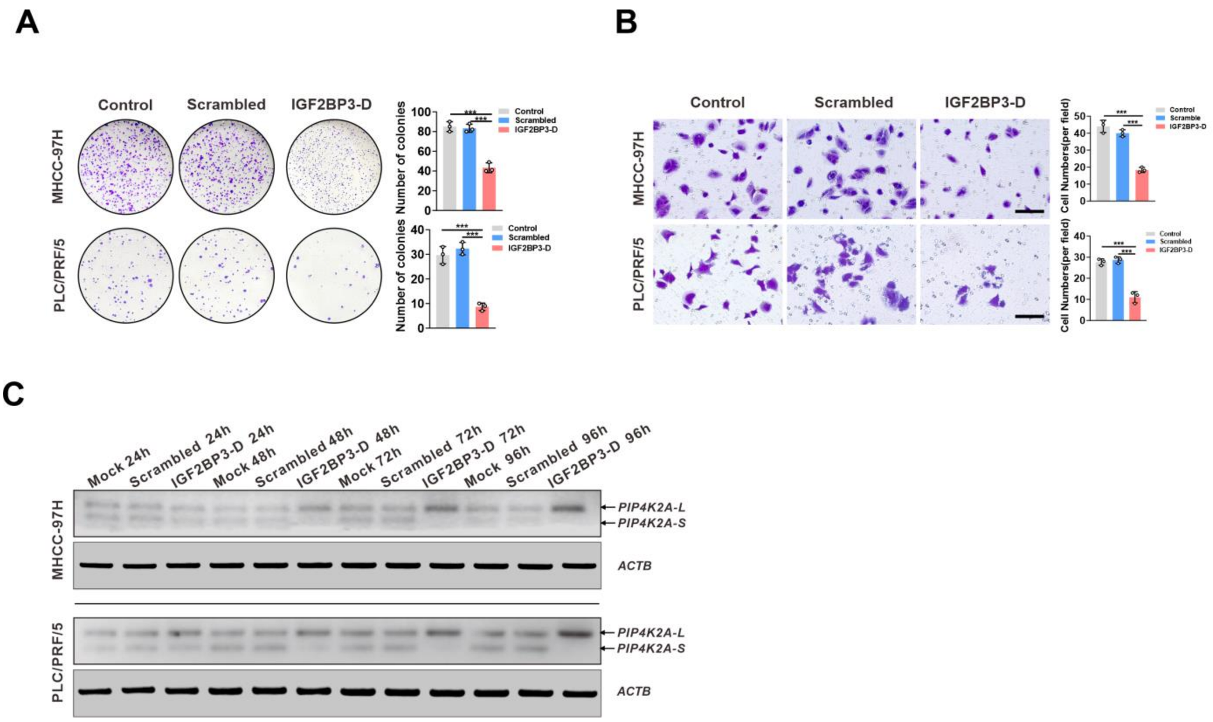

Figure S11. IGF2BP3 decoy oligonucleotides inhibit tumor cell growth and migration in vivo.

Colony formation assays (A) and transwell migration assays (B) of PLC/PRF/5 and MHCC-97H cells transfected with scrambled or IGF2BP3 decoy oligonucleotides. Data are presented as the mean ± SD for three independent experiments. **$p < 0.01$.(C) Quantitative PCR analysis of PIP4K2A-L and PIP4K2A-S in MHCC-97H and PLC/PRF/5 cells transfected with scrambled or IGF2BP3 decoy oligonucleotides.

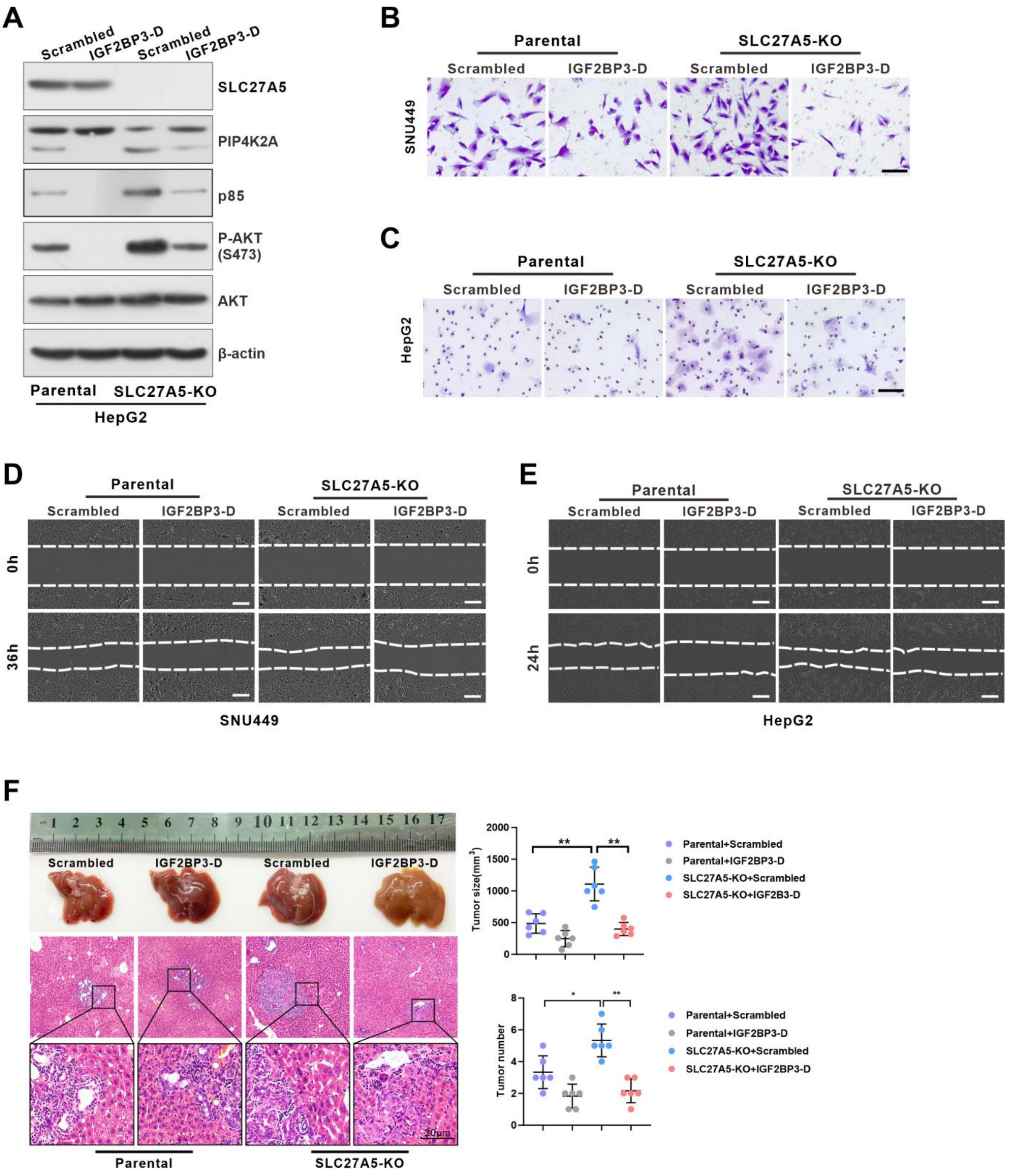

Figure S12. IGF2BP3-D oligonucleotides inhibit SLC27A5 deficiency-induced tumor cell migration.

(A) Western blot analysis of the indicated proteins in SLC27A5-KO cells transfected with scrambled or IGF2BP3 decoy oligonucleotides. (B-E) Cell migration assays (B, C) and wound healing assays (D, E) in SLC27A5-KO cells transfected with scrambled or IGF2BP3 -D oligonucleotides (n = 3 biologically independent samples). (F) Tumor-images and H&E staining. The tumor size and number were calculated. (*$p < 0.05$).

Table S1. Clinical characteristics of HCC patients

	Sex	Age	HBsAg	HBeAg	HBcAb	AFP	HBV DNA	ALT	AST
1	female	50	positive	negative	positive	negative	positive	increase	increase
2	male	59	positive	negative	positive	negative	positive	increase	increase
3	male	35	positive	positive	positive	positive	positive	normal	increase
4	male	59	positive	positive	positive	positive	negative	normal	normal
5	male	40	positive	positive	positive	positive	positive	increase	increase
6	male	36	positive	positive	positive	positive	positive	increase	increase
7	male	44	positive	positive	positive	positive	negative	increase	increase
8	female	55	positive	positive	positive	positive	positive	normal	increase
9	male	60	positive	positive	positive	positive	positive	normal	normal
10	male	52	positive	positive	positive	positive	positive	normal	increase
11	male	63	positive	positive	positive	positive	positive	increase	increase
12	male	38	positive	positive	positive	positive	positive	increase	increase
13	male	43	positive	positive	positive	positive	positive	normal	increase
14	male	71	positive	negative	positive	negative	positive	increase	increase
15	male	47	positive	negative	negative	positive	positive	increase	increase
16	male	65	positive	positive	positive	positive	positive	increase	increase
17	male	44	negative	negative	positive	positive	positive	increase	increase
18	male	37	positive	negative	positive	negative	positive	increase	increase
19	male	46	positive	positive	positive	positive	positive	increase	increase
20	male	57	positive	positive	positive	positive	positive	increase	increase
21	female	40	negative	negative	negative	negative	-	increase	increase
22	male	57	positive	negative	negative	positive	positive	increase	increase
23	male	55	positive	positive	positive	positive	positive	increase	increase
24	male	57	positive	negative	positive	negative	-	increase	increase

25	male	55	positive	positive	positive	negative	positive	increase	increase
26	male	59	positive	positive	positive	negative	positive	normal	normal
27	male	59	positive	positive	positive	negative	positive	normal	normal
28	male	55	positive	positive	negative	negative	negative	normal	normal
29	male	55	positive	positive	positive	positive	positive	increase	increase
30	male	48	positive	negative	positive	positive	-	increase	increase
31	male	63	positive	negative	positive	negative	positive	increase	increase
32	male	57	positive	positive	positive	positive	positive	increase	increase
33	male	62	positive	negative	positive	positive	positive	increase	increase
34	female	64	negative	negative	positive	negative	-	increase	increase
35	male	38	positive	negative	positive	negative	positive	increase	increase
36	male	50	positive	positive	positive	positive	-	increase	increase
37	male	59	positive	negative	positive	negative	positive	increase	increase
38	male	55	positive	positive	positive	negative	negative	increase	increase
39	male	57	positive	positive	positive	positive	negative	increase	increase
40	male	55	positive	positive	positive	negative	positive	increase	increase

Table S2. Relevant basic information and clinicopathological data of tissue microarray cohort (n=67)

Array position	Sex	Age	TNM	metastasis	Tumor free survival (days)	Survival time (days)
**A1,A2**	male	73	T3N0M0	non-metastasis	2372	2372
**A3,A4**	female	52	T1N0M0	non-metastasis	2476	2476
**A5,A6**	male	58	T1N0M0	non-metastasis	2145	2145
**A7,A8**	male	45	T1N0M0	non-metastasis	2675	2675
**A9,A10**	male	47	T1N0M0	non-metastasis	2317	2317
**A11,A12**	female	44	T1N0M0	non-metastasis	1951	1951
**A13,A14**	male	60	T1N0M0	non-metastasis	2092	2092
**A15,A16**	male	32	T1N0M0	non-metastasis	2311	2311
**A17,A18**	female	36	T3N0M0	non-metastasis	252	1388
**B1,B2**	male	40	T2N0M0	non-metastasis	535	979
**B3,B4**	male	49	T2N0M0	metastasis	352	2008
**B5,B6**	male	42	T1N0M0	non-metastasis	2220	2220
**B7,B8**	male	57	T1N0M0	non-metastasis	2247	2247
**B9,B10**	male	51	T1N0M0	non-metastasis	674	943
**B11,B12**	male	45	T1N0M0	non-metastasis	2245	2245
**B13,B14**	female	50	T1N0M0	non-metastasis	2373	2373
**B15,B16**	male	46	T1N0M0	non-metastasis	2242	2242
**B17,B18**	male	42	T3N0M0	non-metastasis	1982	1982
**C1,C2**	female	60	T1N0M0	non-metastasis	2192	2192
**C3,C4**	male	43	T1N0M0	non-metastasis	1980	1980
**C5,C6**	male	49	T3N0M1	metastasis	1116	1973
**C7,C8**	female	45	T1N0M0	non-metastasis	1978	1978
**C9,C10**	male	56	T1N0M0	metastasis	940	1359

**C11,C12**	male	55	T3N0M1	metastasis	34	354
**C13,C14**	male	44	T1N0M0	non-metastasis	2099	2099
**C15,C16**	male	33	T1N0M0	non-metastasis	774	958
**C17,C18**	male	43	T1N0M0	non-metastasis	2077	2077
**D1,D2**	male	59	T3N0M0	non-metastasis	112	440
**D3,D4**	male	49	T3N0M0	non-metastasis	196	296
**D5,D6**	male	48	T1N0M0	non-metastasis	700	700
**D7,D8**	female	50	T2N1M0	non-metastasis	69	1080
**D9,D10**	male	55	T3N0M0	non-metastasis	3114	3114
**D11,D12**	male	45	T2N0M1	metastasis	212	618
**D13,D14**	male	21	T3N0M0	non-metastasis	180	1119
**D15,D16**	male	30	T2N0M0	non-metastasis	336	779
**D17,D18**	female	33	T3N0M1	metastasis	313	454
**E1,E2**	male	56	T3N0M0	non-metastasis	28	143
**E3,E4**	male	53	T2N0M1	metastasis	727	1762
**E5,E6**	male	48	T2N0M0	non-metastasis	1354	1354
**E7,E8**	male	42	T2N0M1	metastasis	136	640
**E9,E10**	male	41	T2N0M0	non-metastasis	3235	3235
**E11,E12**	male	63	T2N0M1	metastasis	924	1083
**E13,E14**	male	39	T2N0M0	non-metastasis	2447	2447
**E15,E16**	male	42	T2N0M1	metastasis	573	647
**E17,E18**	male	47	T2N0M0	non-metastasis	2543	2543
**F1,F2**	male	34	T2N0M0	non-metastasis	2570	2570
**F3,F4**	male	40	T2N0M1	metastasis	2484	2965
**F5,F6**	male	54	T3N0M0	non-metastasis	1045	1390

**F7,F8**	male	55	T3N0M1	metastasis	920	1016
**F9,F10**	male	48	T2N0M1	metastasis	180	355
**F11,F12**	male	64	T3N0M0	non-metastasis	2367	2367
**F13,F14**	male	61	T2N0M1	metastasis	228	506
**F15,F16**	male	41	T3N0M1	metastasis	100	967
**F17,F18**	male	48	T2N0M0	non-metastasis	1356	1681
**G1,G2**	male	30	T2N0M0	non-metastasis	1907	1907
**G3,G4**	female	50	T2N0M1	metastasis	2095	2095
**G5,G6**	male	28	T3N0M0	non-metastasis	588	1169
**G7,G8**	male	48	T2N0M0	non-metastasis	2100	2100
**G9,G10**	male	65	T2N0M0	non-metastasis	2128	2128
**G11,G12**	male	61	T2N0M0	non-metastasis	2109	2109
**G13,G14**	male	59	T2N0M0	non-metastasis	2061	2061
**G15,G16**	male	51	T3N0M0	non-metastasis	2405	2405
**G17,G18**	male	41	T2N0M0	non-metastasis	2264	2264
**H1,H2**	male	73	T2N0M0	non-metastasis	2259	2259
**H3,H4**	male	54	T2N0M0	non-metastasis	2122	2122
**H5,H6**	male	43	T3N0M0	non-metastasis	824	885
**H7,H8**	male	55	T3N0M0	non-metastasis	844	2202
**H9,H10**	male	16	T3N0M0	non-metastasis	934	934
**H11,H12**	male	64	T3N0M0	non-metastasis	2412	2412
**H13,H14**	male	49	T3N0M0	non-metastasis	332	660
**H15,H16**	male	33	T2N0M0	non-metastasis	2345	2345
**H17,H18**	male	70	T2N0M0	non-metastasis	2276	2276
**I1,I2**	male	25	T3N0M0	non-metastasis	132	864

**I3,I4**	male	73	T2N1M1	metastasis	328	444
**I5,I6**	male	47	T2N0M0	non-metastasis	2629	2629
**I7,I8**	male	62	T2N0M1	metastasis	980	1303
**I9,I10**	male	63	T2N0M1	metastasis	1635	1635
**I11,I12**	male	50	T2N0M1	metastasis	558	792
**I13,I14**	male	51	T3N0M0	non-metastasis	367	807
**I15,I16**	male	46	T3N0M0	non-metastasis	126	126
**I17,I18**	male	50	T2N0M1	metastasis	219	422
**J1,J2**	male	59	T1N0M1	metastasis	1443	1443
**J3,J4**	male	41	T3N0M0	non-metastasis	1833	1833
**J5,J6**	male	41	T2N0M0	non-metastasis	793	1237
**J7,J8**	male	59	T1N0M1	metastasis	296	458
**J9,J10**	female	45	T3N0M0	non-metastasis	354	474
**J11,J12**	male	26	T3N0M0	non-metastasis	237	237
**J13,J14**	male	54	T2N0M1	metastasis	1781	1994
**J15,J16**	male	58	T1N0M0	non-metastasis	2297	2297
**J17,J18**	male	52	T2N0M0	non-metastasis	3247	3247

Table S3. Cloning primers sequences and plasmids are used in this study.

**Plasmids**	**Forward (5'-3')**	**Reverse (5'-3')**
pSEB-3Flag-SL<br>C27A5	CGGAAGCTTATGGGTGTCAG<br>GCAACAGTTGGCCTTG	CATGTCGACGAGCCTCCAGGTTCCCT<br>CACA
pAdTrack-TO4-<br>3Flag-SLC27A5<br>(S296A)	TTCATCTATACCGCGGGGAC<br>CACT	AGTGGTCCCCGCGGTATAGATGAA
pcDNA-5myc-I<br>GF2BP3	GGGGTACCATGGGCAACAA<br>ACTGTATATCGGAA	CGGAATTCCCTTCCGTCTTGACTGAG<br>GT
pSEB-3Flag-SL<br>C27A5 1-76aa	AGGGGTACCATGGGTGTCA<br>GGCAACAGTTG	CCCAAGCTTCAGGAGGGTTAGTGCC<br>AGGG
pSEB-3Flag-SL<br>C27A5	AGGGGTACCATGGCACGGC<br>TGCCCCCAGGA	CCCAAGCTTGAGCCTCCAGGTTCCCT<br>CACA

77-690aa		
pcDNA-5myc-IGF2BP3 1-194aa	AGGGGTACCATGGGCAACAAACTGTATATCGGAA	CCGGAATTCCACATGGTTTCTGCTTGGATACG
pcDNA-5myc-IGF2BP3 195-344aa	AGGGGTACCATGGATTTGCCTCTGCGCC	CCGGAATTCCCATGATCTCCTCCTCAGCTTT
pcDNA-5myc-IGF2BP3 345-579aa	AGGGGTACCATGGGCAAGAAAATCAGGGAGTCTTATG	CCGGAATTCCCTTCCGTCTTGACTGAGGTG
pAdTrack-TO4-PIP4K2A	TAGGGTACCATGGCGACCCCCGGCAA	TCCAAGCTTTTACGTCAAGATGTGGCCAAT
pSEB-3Flag-PIP4K2A	TCCAAGCTTACCATGGCGACCCCCGGCAA	CGCGGATCCCGTCAAGATGTGGCCAATAAAGT
shIGF2BP3#1	TGGAAGTGCTGAATGGTGTTGGTTCAAGAGACCAACACCATTCAGCACTTCCTTTTTTC	TCGAGAAAAAAGGAAGTGCTGAATGGTGTTGGTCTCTTGAACCAACACCATTCAGCACTTCCA
shIGF2BP3#2	TGCCAAACCAAAGACAGATTGCTTCAAGAGAGCAATCTGTCTTTGGTTTGGCTTTTTTC	TCGAGAAAAAAGCCAAACCAAAGACAGATTGCTCTCTTGAAGCAATCTGTCTTTGGTTTGGCA
shIGF2BP3#3	TGATTGCTTAACCAACAGATGGTTCAAGAGACCATCTGTTGGTTAAGCAATCTTTTTTC	TCGAGAAAAAAGATTGCTTAACCAACAGATGGTCTCTTGAACCATCTGTTGGTTAAGCAATCA
ShPIP4K2A-S#1	TGATCAAGATTTCCAGTACATTCAAGAGATGTACTGGAAATCTTGATCTTTTTTC	TCGAGAAAAAAGATCAAGATTTCCAGTACATCTCTTGAATGTACTGGAAATCTTGATCA
shPIP4K2A-S#2	TGATCAAGATTTCCAGTACATATTCAAGAGATATGTACTGGAAATCTTGATCTTTTTTC	TCGAGAAAAAAGATCAAGATTTCCAGTACATATCTCTTGAATATGTACTGGAAATCTTGATCA
shPIP4K2A-S#3	TGATTTCCAGTACATAGTGGTTCAAGAGACCACTATGTACTGGAAATCTTTTTTC	TCGAGAAAAAAGATTTCCAGTACATAGTGGTCTCTTGAACCACTATGTACTGGAAATCA

Table S4. Primer sequences are used in this study.

Primers	Sequence(5’-3’)	
**RT-PCR**		
Slc27a5	Forward (5’-3’): TGATGGGACTTGTCGTTGGG	Reverse (5’-3’): TATGTGTCCGGTCCTCTGGT
β-actin	Forward (5'-3'): AGGCCAACCGCGAGAAGATGACC	Reverse (5’-3’): GAAGTCCAGGGCGACGTAGCAC
PIP4K2A	Forward (5'-3'): CAAAAATAAAGGTGGACAATC	Reverse (5’-3’): AGACAAACGGTGGCTGAA
PIP4K2A-L	Forward (5'-3'): TGATCAAGATTTCCAGAATTCCC	RReverse (5’-3’): CATGACATTCCACTATGTACTGGTG
PIP4K2A-S	Forward (5'-3'): TTGATGATCAAGATTTCCAGTACATAG	Reverse (5’-3’): CACTGTAGAGCCCTTTAAGTCGT
Pip4k2a	Forward (5'-3'): GAATGTGTTCAGCCACCG	Reverse (5’-3’): TTGCATCGTAATGAGTAAGGA
Pip4k2a-L	Forward (5'-3'): TGTGGAGCGAGCAGAGCA	Reverse (5’-3’): AGGAGCCAAGGGTGGTGAG
Pip4k2a-S	Forward (5'-3'): ACCACCAGGGCTCGACAGT	Reverse (5’-3’): GGCGCATCTCGACATCCT
IgForward (5'-3')2bp3	Forward (5'-3'): CAAGTTCCACCCCCTCCA	Reverse (5’-3’): GGGCGACACTGCTTTTTCT
Cmyc	Forward (5'-3'): AGAGAAGCTGGCCTCCTACC	Reverse (5’-3’): CGTCGAGGAGAGCAGAGAAT
E-cadherin	Forward (5'-3'): AATTGCTCACATTTCCCAACTC	Reverse (5’-3’): CTCTGTCACCTTCAGCCATCC
Pdl1	Forward (5'-3'): TGTGGCATCCAAGATACAAACTCAAAG	Reverse (5’-3’): TCCTCCTCTGCTTTCGCCAGGTTC
**IF-Fish mRNA probe**		
PIP4K2A	Cy3-GGACACATACTGACCGAAGTG	
**IGF2BP3 decoy sequences**		
Decoy	UCGGCUAUCCACACACACUU	
Scramble	AAAAAGCUGCUCCUCUCCUC	

Table S5. Antibodies are used in this study.

Antibodies	Species	application	Concentration	Company source	Cat #
SLC27A5	Mouse	WB	1:1000	Gene Tex	GTX60688
SLC27A5	Mouse	IP	1:100	Novus Biologicals	NBP2-37412
SLC27A5	Mouse	IF	1:500	Gene Tex	GTX60688
SLC27A5	Mouse	IHC	1:500	Gene Tex	GTX60688
P-AKT(s473)	Rabbit	WB	1:1000	Bioworld Technology	BS4007
P-AKT(s473)	Rabbit	IF	1:500	Bioworld Technology	BS4007
P-AKT(s473)	Rabbit	IHC	1:200	Bioworld Technology	BS4007
AKT	Rabbit	WB	1:1000	Bioworld Technology	AP0059
P85	Rabbit	WB	1:1000	Cell Signaling Technology	4257
P85	Rabbit	IF	1:500	Cell Signaling Technology	4257
P85	Rabbit	IHC	1:500	Cell Signaling Technology	4257
HA-tag	Mouse	IP	1:5000	Invitrogen	26183
P110 alpha	Rabbit	WB	1:1000	Cell Signaling Technology	4249T
P110 Beta	Mouse	WB	1:5000	Proteintech	67121-1-Ig
PIP4K2A	Rabbit	WB	1:1000	Proteintech	12469-1-AP
c-MYC	Rabbit	WB	1:5000	Proteintech	10828-1-AP
Flag-tag	Mouse	IP	1:5000	Sigma-Aldrich	F3165
Flag-tag	Mouse	IF	1:10000	Sigma-Aldrich	F3165
IGF2BP3	Rabbit	WB	1:5000	Proteintech	14642-1-AP
IGF2BP3	Rabbit	IP	1:2000	Proteintech	14642-1-AP
IGF2BP3	Rabbit	IF	1:50	Proteintech	14642-1-AP
Myc-Tag	Rabbit	IP	1:2000	Cell Signaling Technology	2272S
PDL1	Mouse	WB	1:5000	Proteintech	66248-1-Ig
MMP2	Rabbit	IHC	1:500	Gene Tex	GTX104577
MMP9	Rabbit	IHC	1:400	Cell Signaling Technology	13667T
E-cadherin	Rabbit	IHC	1:500	Abcam	ab40772
Vimentin	Rabbit	IHC	1:150	Cohesion Biosciences	CPA2228
GAPDH	Mouse	WB	1:1000	Beyotime	AF0006
β-actin	Mouse	WB	1:1000	ZSGB-BIO	TA-09
Goat anti-rabbit IgG/TRITC, secondary	Goat	IF	1:50	ZSGB-BIO	ZF-0316
Goat anti-mouse IgG/ TRITC, secondary	Goat	IF	1:50	ZSGB-BIO	ZF-0313
Goat anti-rabbit IgG/FITC, secondary	Goat	IF	1:50	ZSGB-BIO	ZF-0311
PIP4K2A-S	Rabbit	WB	1:2000	ABclonal	WG-06041
PIP4K2A-S	Rabbit	IHC	1:200	ABclonal	WG-06041
PIP4K2A-L	Rabbit	WB	1:2000	ABclonal	WG-06040
PIP4K2A-L	Rabbit	IHC	1:200	ABclonal	WG-06040
